# Fuzheng Nizeng Decoction regulated ferroptosis and endoplasmic reticulum stress in the treatment of gastric precancerous lesions: A mechanistic study based on metabolomics coupled with transcriptomics

**DOI:** 10.3389/fphar.2022.1066244

**Published:** 2022-11-23

**Authors:** Ying-Ming Chu, Ting-Xin Wang, Xiao-Fen Jia, Yao Yang, Zong-Ming Shi, Guang-Hui Cui, Qiu-Yue Huang, Hui Ye, Xue-Zhi Zhang

**Affiliations:** ^1^ Department of Integrated Traditional Chinese and Western Medicine, Peking University First Hospital, Institute of Integrated Traditional Chinese and Western Medicine, Peking University, Beijing, China; ^2^ Institute (College) of Integrative Medicine, Dalian Medical University, Dalian, China

**Keywords:** ferroptosis regulation, natural medicine, gastric precancerous lesions, endoplasmic reticulum stress, metabolism, transcriptomics

## Abstract

**Background:** Fuzheng Nizeng Decoction (FZNZ) has a history of decades in gastric precancerous lesions (GPL) treatment, which has shown clear clinical efficacy. Blocking GPL is a key measure to reduce the incidence of gastric cancer (GC). Therefore, we aim to investigate the mechanism of FZNZ-induced ferroptosis and endoplasmic reticulum (ER) in MNNG-induced gastric precancerous lesion (MC) cells, which has been rarely studied in Traditional Chinese Medicine (TCM).

**Methods:** First, CCK8 and lactate dehydrogenase assays were conducted to study the potential effect of FZNZ on MC cells. Second, combined transcriptomic and metabolomic analysis were used to explore the effect and mechanism of FZNZ. Functionally, the occurrence of ferroptosis was assessed by transmission electron microscopy morphological observation and measurement of ferrous iron levels, lipid peroxidation, and glutathione levels. Finally, the expression levels of mRNAs or proteins related to ferroptosis and ER stress were determined by qPCR or western blot assays, respectively.

**Results:** FZNZ inhibited MC cells viability and induced cell death. By metabolomics coupled with transcriptomics analysis, we found that the mechanism of FZNZ treatment induced ferroptosis and was related to glutathione metabolism and ER stress. We then, for the first time, found that FZNZ induced ferroptosis, which contributed to an increase in intracellular ferrous iron, reactive oxygen species, and malondialdehyde and a decrease in glutathione. Meanwhile, the protein level of glutathione peroxidase 4 (GPX4) was decreased. The mRNA levels of ATF3/CHOP/CHAC1, which are related to ferroptosis and ER stress, were also upregulated.

**Conclusion:** Our results elaborate that FZNZ could induce ferroptosis and ER stress in MC cells, and reduce GPX4/GSH. ATF3/CHOP/CHAC1 may play a crosstalk role, which provides a new molecular mechanism for the treatment of GPL.

## Graphical Abstract

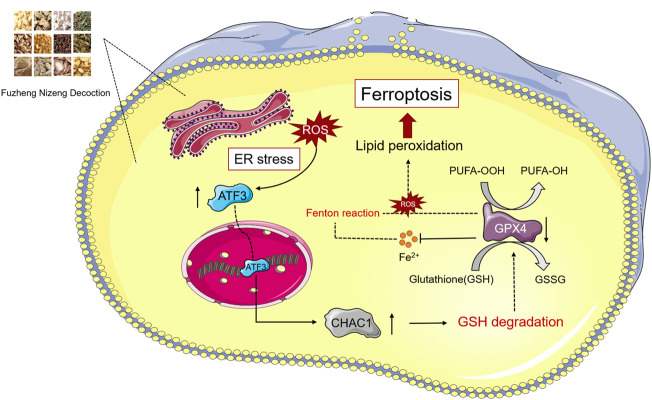

## Introduction

According to the Correa model, the malignant development of normal gastric mucosa into gastric cancer is a long-term stage, especially in the three stages of “chronic atrophic gastritis - intestinal metaplasia - intraepithelial neoplasia” (Correa, 1988). Therefore, three stages are termed gastric precancerous lesions (GPL), which are well-known risk factors for the development of intestinal-type gastric adenocarcinomas (Fu et al., 2022). However, at present, there is no effective medicine for GPL treatment in modern medicine. The main treatment measures are eliminating pathogenic factors and so on, such as eradication of *Helicobacter pylori*, anti-bile reflux therapy, and endoscopic therapy (Guo et al., 2022). Therefore, it is urgent to find more effective drugs for GPL treatment. Regulated cell death (RCD) is controlled by specific signal transduction pathways (Peng et al., 2022), which can be modulated by pharmacological or genetic interventions (Galluzzi et al., 2018). Ferroptosis is an iron-dependent form of RCD driven by excessive lipid peroxidation and subsequent plasma membrane rupture (Dixon et al., 2012; Stockwell et al., 2017). A previous study identified ferroptosis in gastric cancer progression (Hao et al., 2017), suggesting that ferroptosis could be a suitable target for treatment of GPL.

Ferroptosis has been linked to many human diseases, such as neurodegenerative diseases (Dionísio et al., 2021), renal degeneration (Stockwell et al., 2017) and metabolic disease (Ajoolabady et al., 2021). The GSH/GPX4 axis has been recognized as the mainstay in ferroptosis control (Friedmann Angeli et al., 2014). Depletion of glutathione (GSH) results in inactivation of glutathione peroxidase 4 (GPX4), which leads to the accumulation of reactive oxygen species (ROS) in lipid peroxidation and subsequent ferroptosis (Zheng and Conrad, 2020). Ferroptosis is mainly controlled by the GSH redox system (Xiong et al., 2021), which means that whatever affects GSH metabolism may also indirectly regulates ferroptosis. Emerging evidence suggests that endoplasmic reticulum (ER) stress plays an essential role in ferroptosis through activation of the activating transcription factor 3 (ATF3)-C/EBP homologous protein (CHOP) cascade (Lee et al., 2018) and subsequent activation of cation transport regulator 1 (CHAC1) (Hamano et al., 2020). As a constituent of ER stress, the increase in CHAC1 leads to the degradation of GSH into 5-oxoproline and cysteinyl-glycine (Kumar et al., 2012) and serves as a pharmacodynamic marker of ferroptosis (McCullough et al., 2001).

Many pharmacological studies have found that Traditional Chinese Medicine (TCM) plays an important role in treating GPL due to its multitarget characteristics (Xu et al., 2018; Tong et al., 2021). The pathogenesis of GPL can be summarized as “Qi deficiency, blood stasis and toxin” in TCM. Fuzheng Nizeng Decoction (FZNZ) is derived from the classic formula Liujunzi Decoction. Our previous clinical studies have proved that FZNZ has achieved good clinical efficacy in GPL treatment (Xin et al., 2021a). However, the mechanism of FZNZ in the treatment of GPL remains to be further studied.

**FIGURE 1:**
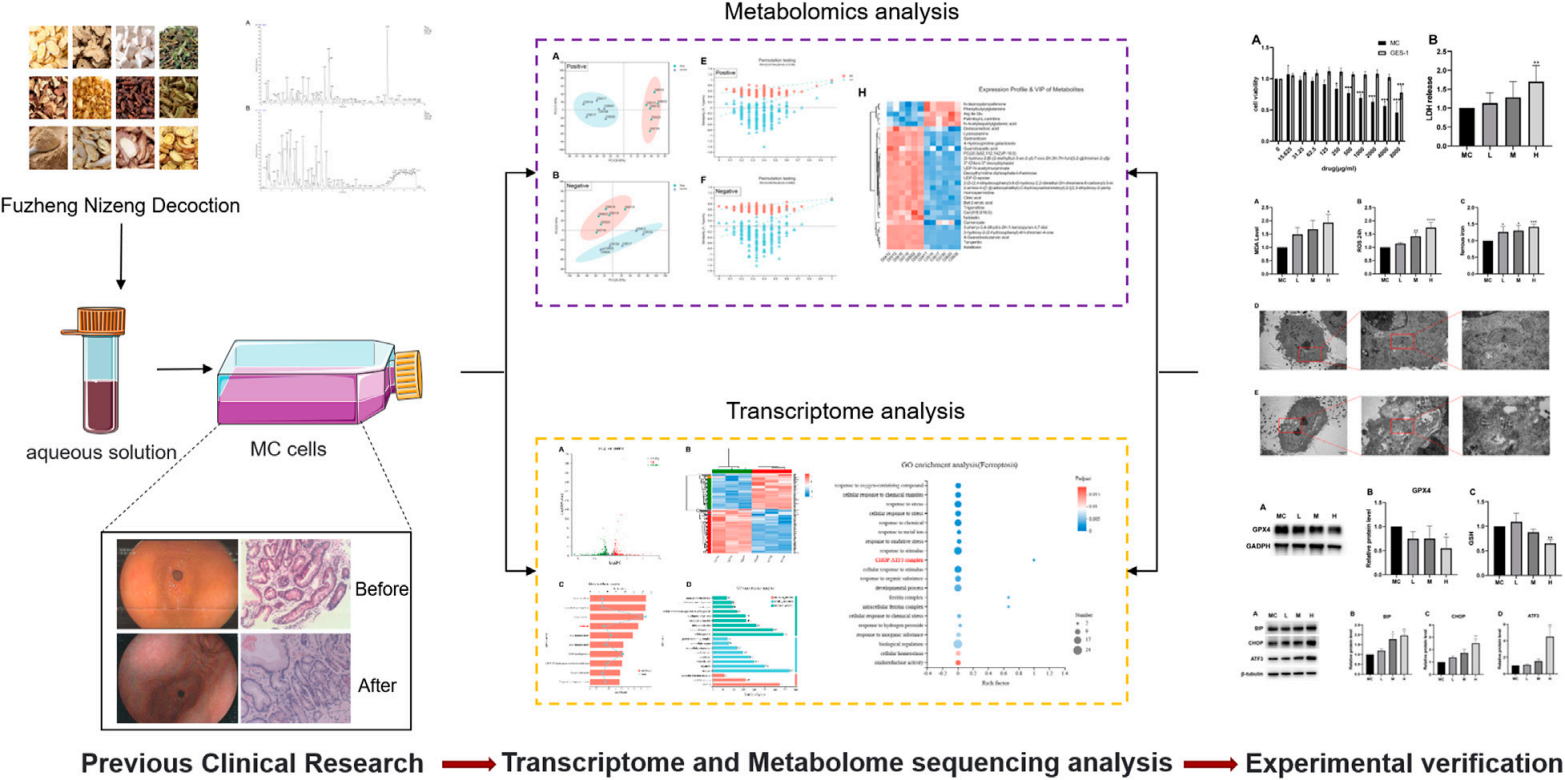
Flowchart of experimental design. Based on the clinical efficacy of FZNZ, the mechanism of action of FZNZ was explored by combined analysis of transcriptome and metabolome and verified *in vitro*. A representative gastroscopy and pathological results are given in figure: Before treatment: (gastric antrum) pyloric gland type gastric mucosa mild chronic inflammation, one part of the stomach severe atrophic gastritis, severe intestinal metaplasia; After treatment: (gastric antrum, gastric Angle) mild chronic gastritis.

This study combined metabolomics and transcriptomics *in vitro* to provide new insights for clinical intervention and GPL treatment, as well as to elucidate the mechanism (Figure 1).

## Materials and methods

### Drug preparation

FuZheng NiZeng Decoction component: Astragalus mongholicus Bunge [Fabaceae; Astragali radix] 15 g, Atractylodes macrocephala Koidz. [Asteraceae; Atractylodis macrocephalae rhizoma] 10 g, Glycyrrhiza uralensis Fisch. ex DC. [Fabaceae; Glycyrrhizae radix rhizoma] 5 g, Citrus × aurantium L. [Rutaceae; Citri reticulatae pericarpium] 10 g, Cyperus rotundus L. [Cyperaceae; Cyperi rhizoma] 15 g, Panax notoginseng (Burkill) F.H.Chen [Araliaceae; Notoginseng radix et rhizoma] 3 g, Pinellia ternata (Thunb.) Makino [Araceae; Pinelliae rhizoma] 5 g, Poria Cocos (Schw.) Wolf. [Polyporaceae; Poria] 10 g, Curcuma aromatica Salisb. [Zingiberaceae; Curcumae radix] 12 g, Salvia miltiorrhiza Bunge [Lamiaceae; Salviae miltiorrhizae radix et rhizoma] 10 g, Actinidia chinensis Planch. [Actinidiaceae; Radix actinidiae chinensis] 15 g, Scutellaria barbata D. Don [Lamiaceae; Scutellariae barbatae herba] 15 g and Solanum lyratum Thunb. [Solanaceae; Herba Solani Lyrati] 15 g. Each 140 g of botanical drugs contained are listed in Table 1. All botanical drugs were purchased from Beijing Jinxiang Fuxing Medicine Co. Baita Temple Pharmacy (Beijing, China) and reviewed by pharmacists of Peking University First Hospital. A total of 700 g of five decoctions were soaked in water for 4 h, decocted for 2.5 h, precipitated, filtered for 8 h, concentrated for 30 min, and prepared into 100 ml ointments. Finally, the extract was prepared into a mother solution at a concentration of 1 g/ml using RPMI 1640 medium and stored at -20°C (Xu et al., 2018; Fang et al., 2020; Tong et al., 2021). When used in cell experiments, RPMI-1640 culture medium was used to adjust the corresponding concentration, and after ultrasonic dissolution, it was filtered through 0.22 μm sterile.

**TABLE 1:** Composition of FZNZ.

Drug name	Chinese name	Area	Plant part	Mass (g)
Astragalus mongholicus Bunge [Fabaceae; Astragali radix]	Huang Qi	Inner Mongolia	root	15
Atractylodes macrocephala Koidz. [Asteraceae; Atractylodis macrocephalae rhizoma]	Bai Zhu	Anhui	rhizome	10
Glycyrrhiza uralensis Fisch. ex DC. [Fabaceae; Glycyrrhizae radix rhizoma]	Gan Cao	Gansu	rhizome	5
Citrus × aurantium L. [Rutaceae; Citri reticulatae pericarpium]	Chen Pi	Sichuan	fruit peel	10
Cyperus rotundus L. [Cyperaceae; Cyperi rhizoma]	Xiang Fu	Henan	rhizome	15
Panax notoginseng (Burkill) F.H.Chen [Araliaceae; Notoginseng radix et rhizoma]	San Qi	Yunnan	root	3
Pinellia ternata (Thunb.) Makino [Araceae; Pinelliae rhizoma]	Ban Xia	Sichuan	tuber	5
Poria Cocos (Schw.) Wolf. [Polyporaceae; Poria]	Fu Ling	Anhui	sclerotium	10
Curcuma aromatica Salisb. [Zingiberaceae; Curcumae radix]	Yu Jin	Guangxi	tuber	12
Salvia miltiorrhiza Bunge [Lamiaceae; Salviae miltiorrhizae radix et rhizoma]	Dan Shen	Shandong	root	10
Actinidia chinensis Planch. [Actinidiaceae; Radix actinidiae chinensis]	Teng Ligen	Zhejiang	root	15
Scutellaria barbata D.Don [Lamiaceae; Scutellariae barbatae herba]	Ban Zhilian	Jiangsu	whole plant	15
Solanum lyratum Thunb. [Solanaceae; Herba Solani Lyrati]	Bai Ying	Jiangsu	whole plant	15

### UHPLC-orbitrap-MS analysis

The phytochemical profile of FZNZ was carried out an UHPLC system (Vanquish, Thermo Fisher Scientific) with a Waters UPLC ethylene bridged hybrid (BEH) C18 column (1.7 μm 2.1*100 mm). The flow rate was 0.5 ml/min and sample inject volume was 5 μL. The mobile phase consisted of 0.1% formic acid in water (A) and 0.1% formic acid in acetonitrile (B). The multi-step linear elution gradient program was as follows: 0–11 min, 85–25% A; 11–12 min, 25–2% A; 12–14 min, 2–2% A; 14–14.1 min, 2–85% A; 14.1–16 min, 85–85% A. The data were collected in both positive and negative ion modes. During each acquisition cycle, the mass range was from 100 to 1,500, and the top four of every cycle were screened and the corresponding MS/MS data were further acquired. Sheath gas flow rate: 35 Arb, Aux gas flow rate: 15 Arb, Ion Transfer Tube Temp: 350°C, Vaporizer Temp: 350°C, Full ms resolution: 60,000, MS/MS resolution: 15,000, Collision energy: 16/32/48 in NCE mode, Spray Voltage: 5.5 kV (positive) or -4 kV (negative). All data acquisition and analyses were performed using an Orbitrap Exploris 120 mass spectrometer coupled with Xcalibur software based on the IDA acquisition mode. Chemical constituents identified of FZNZ were in Supplementary Table S1.

### Cell culture and reagents

Human immortalized gastric epithelial GES-1 cells were purchased from Procell Life Science & Technology Co. (Wuhan, China). MC cells were identified and endowed by Beijing University of Traditional Chinese Medicine, and the related experiments have been specifically described in previous studies (Wang et al., 2021). All cells were cultured in RPMI 1640 medium (Gibco) containing 10% fetal bovine serum (Gibco), 100 U/mL penicillin and 100 μg/ml streptomycin. For culture, all cells were grown in a humidified incubator containing 5% CO2 and maintained at 37°C.

### Transcriptome analysis

MC cells seeded in 100 mm culture dishes were treated with high concentration FZNZ for 24 h and then total RNA was then extracted with TRIzol reagent (Invitrogen, United States ). Among them, the control group was labeled as C0308, C0102, C0205, and the drug treatment group was labeled as D0101, D0204, D0307. The concentration and purity of the RNA were examined using Nanodrop 2000, RNA integrity was examined by agarose gel electrophoresis, and RIN values were determined by Agilent2100. Eukaryotic mRNA sequencing was performed using the Illumina NovaSeq 6,000 sequencing platform. The corresponding sequencing data has been uploaded to the SRA database (PRGNA895170).

### Sample preparation for metabolomic analysis

MC cells were divided into two groups: the control group (RPMI-1640 culture medium, Control) and the drug group (FZNZ 4000 μg/ml treatment for 24 h, Drug). All samples were placed into a centrifuge tube, and a 6 mm diameter grinding bead was added along with 400 µL of extraction solution (methanol: water = 4:1 (v:v)) containing 0.02 mg/ml internal standard (L-2-chlorophenylalanine). Subsequently, the frozen tissue grinder was ground for 6 min (-10°C, 50 Hz), and low-temperature ultrasonic extraction was performed for 30 min (5°C, 40 kHz). Finally, the samples were left at -20°C for 30 min and centrifuged for 15 min (13,000 g, 4°C), and the supernatant was pipetted into the injection vial with an internal cannula for analysis on the machine. In addition, 20 µL of supernatant was pipetted separately for each sample, mixed and used as quality control (QC) samples.

### LC‒MS metabolomics analysis

The instrumental platform was used ultrahigh-performance liquid chromatography tandem Fourier transform spectrometry (UHPLC-QExactiveHF-X) system (Thermo Fisher) for this LC‒MS analysis. Among the chromatographic conditions, the column was ACQUITYUPLCHSST3 (100 mm × 2.1 mm d, 1.8 µm; Waters, Milford, United States ); The injection volume was 4 μL, and the column temperature was 40°C for mass spectrometry conditions. The samples were ionized by electrospray ionization, and the mass spectrometric signals were collected in positive and negative ion scanning modes.

### Transmission electron microscopy

Well-grown MC cells were cultured in 60 mm dishes, and after adding RPMI-1640 and a high concentration of FZNZ for 24 h, they were digested with EDTA-free trypsin and washed twice with PBS at room temperature. Samples were placed in electron microscope fixative glutaraldehyde at 4°C overnight. The cells were immobilized with osmic acid, dehydrated with ethanol, infiltrated, embedded in epoxy resin, ultrathin sectioned, stained with uranium and lead, observed, and photographed under a transmission electron microscope.

### Cell viability assay

FZNZ were added to the drug groups with different concentrations, and RPMI-1640 was added in the blank and control groups. The cell culture process was performed at 37°C and 5% carbon dioxide. After 24 h of treatment with the corresponding drugs, each well was added 100 μL CCK8 solution (Dojindo Laboratories, Japan) and incubated for 1 h. The microplate reader was set at 450 nm to measure the OD value of each sample.

### Detection of lactate dehydrogenase (LDH)

The release of LDH was assessed using the Lactate Dehydrogenase Release Assay Kit (Nanjing Jiancheng, catalog number: A020-2-2). In short, MC cells were cultured in 6-well plates, and FZNZ was added to cells and incubated at 37°C for 24 h. Then, the supernatant was added to the LDH reaction mixture and then transferred to a new 96-well plate to detect the absorbance at 450 nm as directed by the manufacturer.

### Flow cytometry

MC cells were treated with different concentration FZNZ or with RPMI 1640 for 24 h, washed twice in PBS, collected with trypsin without EDTA (Gibco, United States ). According to the instructions of the Annexin V-FITC Apoptosis Detection Kit (KeyGEN BioTECH, KGA108, China), 5 μL FITC and PI were then added to 400 μL binding buffer solution. After mixing for 10–15 min in the dark, the cells were subjected to a BD FACSCalibur flow cytometer. The analyses of the apoptosis rate were performed using FlowJo_V10.

### Determination of malondialdehyde (MDA) levels

After 24 h of drug treatment at the indicated concentrations, cell extracts from the indicated cells were collected and prepared according to the manufacturer. MDA levels were detected by a Cell Malondialdehyde assay kit (Najing Jiancheng, catalog number: A003-4-1). Its maximum absorption wavelength is 535 nm.

### Measurement of glutathione (GSH)

A commercial reduced glutathione kit (Najing Jiancheng, catalog number: A006-2-1) was used for the assay. Cells in six-cell plates were treated with FZNZ for 24 h, scraped into 300 μL PBS and then lysed by sonication. After centrifugation, the supernatant was aspirated, and the concentration was determined according to the reagent instructions. The protein concentration was also measured using the BCA method. Absorbance at 405 nm was measured, and the GSH content was calculated with reference to the protein standard.

### Measurements of iron assay and ROS

Assay kits were used to measure iron concentrations and ROS in cells. The cells were encased in black 96-well plates and stimulated with the drug for 24 h. FerroOrange (Dojindo Laboratories, Japan) dispersed in 1,640 was added to the cells and incubated at 37°C for 30 min. A fluorescence microplate reader was used to read at Ex: 543 nm and Em: 580 nm. For ROS, cells were incubated with DCFH-DA (Najing Jiancheng, catalog number: E004-1-1) at 37°C for 30 min. After 0.5 h of incubation, the remaining dye was washed off with PBS. The generated fluorescence intensity was measured with a fluorometer at Ex: 488 nm and Em: 525 nm.

### Quantitative real-time polymerase chain reaction (qRT‒PCR)

Total RNA in cells was extracted using TRIzol reagent. Aftert RNA concentrations were determined, cDNA was synthesized by using the cDNA Synthesis Kit (Takara, Japan) according to the manufacturer. The relative expression of mRNA was calculated by the 2^−△△Ct^ method. The sequences of the primers for qRT‒PCR are presented in Table 2. GAPDH served as the reference control gene.

**TABLE 2:** Primer sequences.

Primer name	Sequence (5'→3′)
ATF3 fwd	CCT​CTG​CGC​TGG​AAT​CAG​TC
ATF3 rev	TTC​TTT​CTC​GTC​GCC​TCT​TTT​T
DDIT3 fwd	GGA​AAC​AGA​GTG​GTC​ATT​CCC
DDIT3 rev	CTG​CTT​GAG​CCG​TTC​ATT​CTC
CHAC1 fwd	GAA​CCC​TGG​TTA​CCT​GGG​C
CHAC1 rev	CGC​AGC​AAG​TAT​TCA​AGG​TTG​T
GAPDH fwd	ATG​ATG​ACA​TCA​AGA​AGG​TGG​TGA
GAPDH rev	GTC​ATA​CCA​GGA​AAT​GAG​CTT​GAC​A

### Western blot

Protein concentrations were detected using a BCA kit in RIPA lysis buffer containing protease inhibitors and PMSF (Beyotime Biotechnology, Shanghai, China). Electrophoresis was performed using a 4–12% precast gel (LABLEAD, Beijing, China) and transferred to PVDF membranes (Millipore, United States ). The membranes were closed at room temperature for 1 h in 5% BSA and then left overnight at 4 °C containing the corresponding antibodies. After applying TBST to wash the membrane, the secondary antibodies were left at room temperature for 1 h. Pictures of the membranes were obtained using a BIO-RAD instrument (Bio-Rad Laboratories, Hercules, United States ). The following antibodies were used: ATF3 (Santa Cruz, sc-81189, 1:500), GPX4 (Abclonal, A11243, 1:1,000), BIP (Santa Cruz, sc-166490, 1:500), and CHOP (Proteintech, 15204-1-AP, 1:1,000).

### Statistical analysis

Repeat at least three times for each experiment. Data are expressed as the mean ± SD or SEM. One-way analysis of variance (ANOVA) and Tukey’s multiple comparison test were used to compare significant differences. Column graphs were drawn using GraphPad Prism 8.0 software. *p*-value < 0.05 was considered statistically significant.

## Results

### Identification of compounds in FZNZ

**FIGURE 2:**
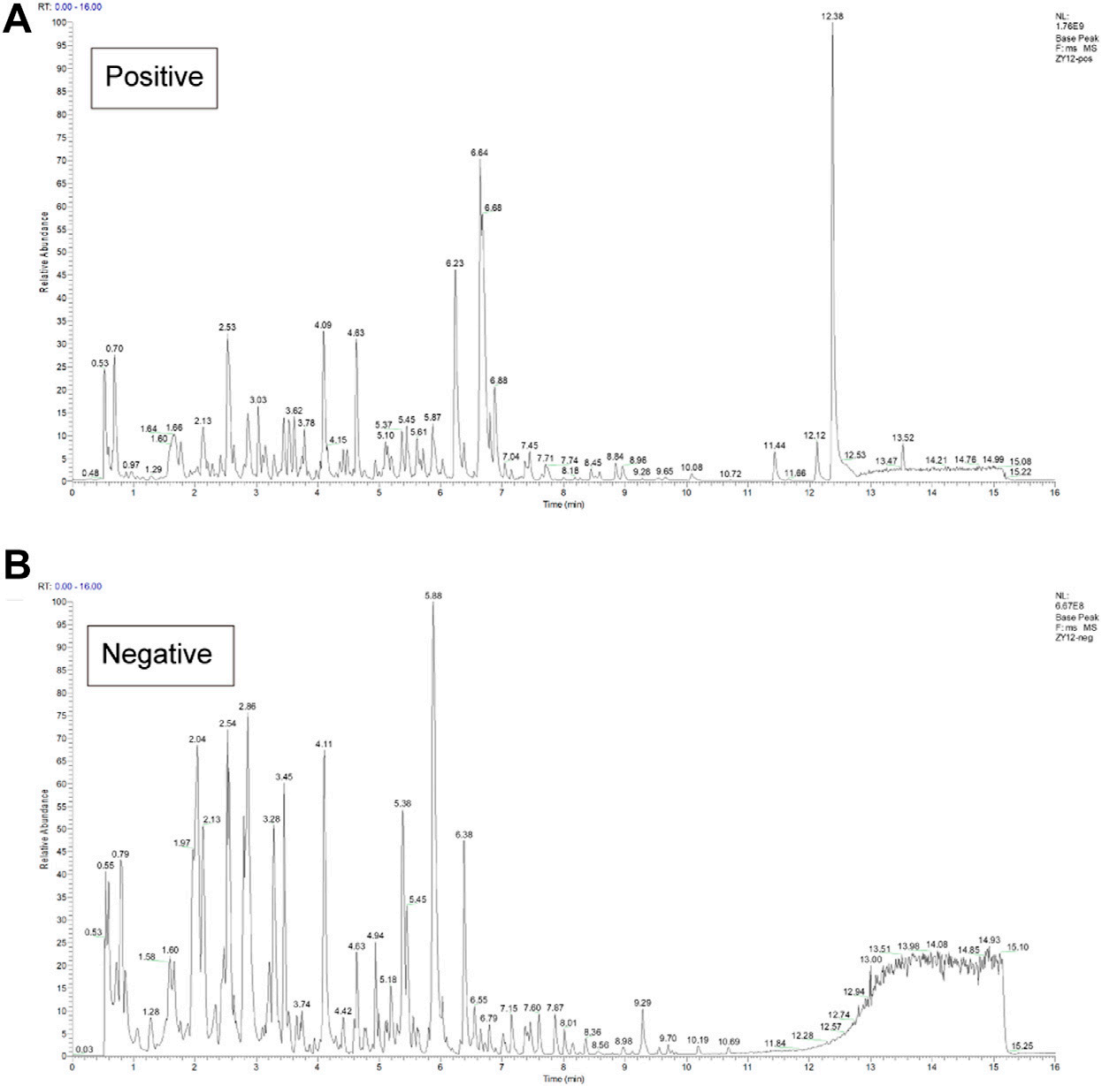
Chemical characterization of the FZNZ was determined by UPLC-QE-MS **(A)** Total ion flow diagram of FZNZ in a positive mode **(B)** Total ion flow diagram of FZNZ in a negative mode.

The composition of FZNZ samples is listed in Table 1. This study was performed compound using UPLC-QE-MS in positive and negative ion modes for detection (Figure 2) and Xcalibur2.1 workstation for spectral processing. Compound identification was performed by scoring the UPLC mass spectra for similarity comparison with the database after a series of optimization processes. Then, we used XCMS to search the library. Among them, 331 compounds were detected by positive ions, and 158 compounds were detected by negative ions, of which all 37 compounds were detected. (Supplementary Table S1).

### FZNZ compromised MC cell viability

**FIGURE 3:**
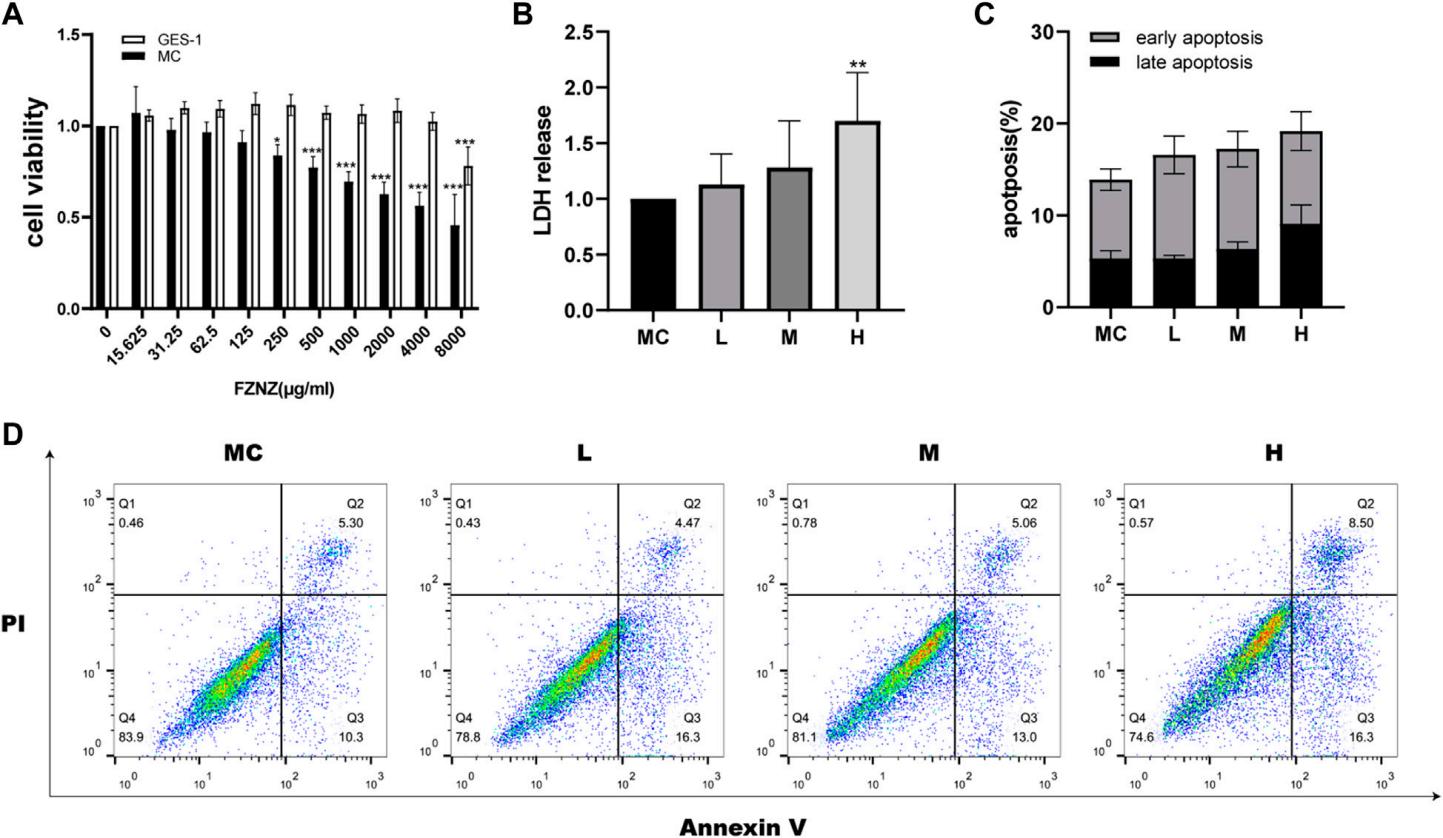
FZNZ inhibited MC cell viability and induced cell death. **(A)** MC and GES-1 cells were incubated with various concentrations of FZNZ for 24 h. Cell viability was determined by CCK8 assay. **(B)** MC cells were treated with different concentrations of FZNZ for 24 h, and cytotoxicity were evaluated by LDH assay. **(C)**. The percentage of apoptotic MC cells. **(D)**. Representative dot-plots illustrated the apoptotic status of MC cells. Data are shown as the mean ± SD or SEM; N = 3–5/group (*p < 0.05, **p < 0.01, ***p < 0.001).

To investigate the effect of FZNZ on GPL, normal gastric epithelial GES-1 cells and MC cells were treated with different concentrations of FZNZ for 24 h, and cell growth potential was detected by the CCK-8 method. When the drug concentration reached 250 μg/ml, the viability of MC cells showed a decrease; when it reached 8,000 μg/ml, the viability of GES-1 cells showed a decrease (Figure 3A). Therefore, we chose 250, 1,000, and 4,000 μg/ml as the low (L), medium (M), and high (H) dose groups for drug treatment of MC cells. To further understand the therapeutic basis of FZNZ, we measured the level of LDH release, as cytoplasmic LDH is rapidly released into cell culture media when the plasma membrane and cell membrane are damaged. The cellular cytotoxicity increased in MC cells with increasing concentrations of FZNZ and was most pronounced in the high-dose group, which suggested cell membrane rupture death (Figure 3B).

At the same time, in order to explore the cause of the cytotoxicity of FZNZ on MC cells, flow cytometry was used to detect apoptosis. With the increase of drug concentration, the apoptosis showed an increasing trend, but there was no significant statistical difference (Figures 3C,D). To explore the way of cell death, we subsequently performed transcriptome and metabolome sequencing analysis.

### Transcriptional changes in MC cells treated by FZNZ

**FIGURE 4:**
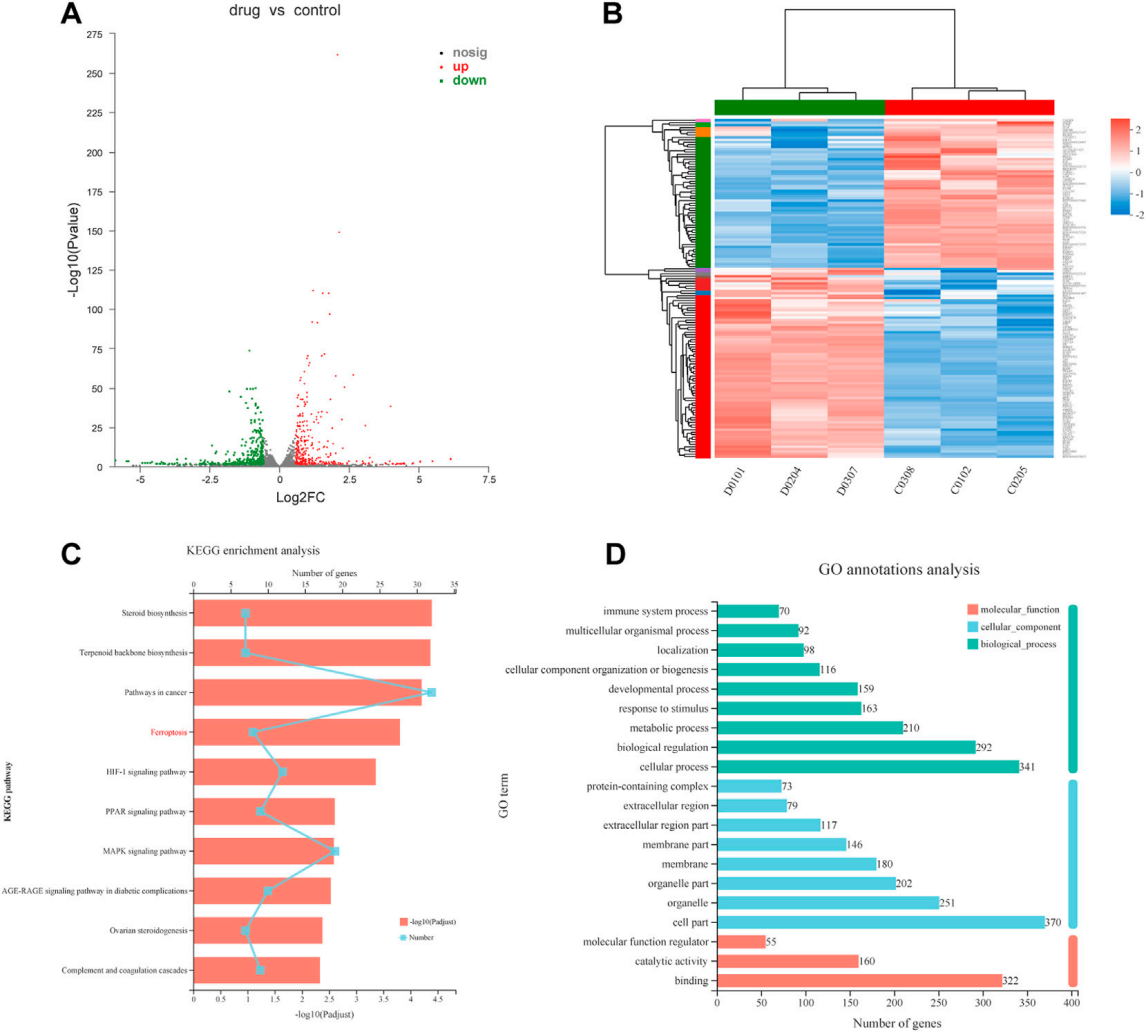
Transcriptional changes in MC cells **(A)**Volcano plot showing differentially expressed genes in MC cells between the control group and FZNZ group. Down-regulation is depicted in green, while up-regulation is depicted in red. FZNZ, 4,000 μg/ml. **(B)** Heatmap showing the cluster of differentials in FZNZ treatment and control group. Up- and downregulation are represented in red and blue, respectively. FZNZ, 4,000 μg/ml. **(C)** KEGG analysis **(D)**GO analysis. The *y*-axis represents the main pathways.

To explore the causes of drug-induced cell death, we performed a transcriptome analysis of the high-dose drug group with MC cells. Based on the expression quantification results, differential expressed genes (DEGs) analysis was performed using DESeq2 with a screening threshold of |log2FC| ≥ 0.585 and *p*-value < 0.05. There were 826 DEGs, of which 381 were upregulated and 445 were downregulated (Figure 4A). The heatmap illustrates that under the effect of drugs, the clustering of genes was apparent (Figure 4B). The results were analyzed by pathway enrichment. Figure 4C listed the top 10 pathways analyzed by KEGG, with the highest number of enriched genes was Pathways in cancer, suggesting possible intervention in cancer progression in response to drug action. Interestingly, the ferroptosis phenotype was also enriched, and we speculated that the FZNZ-induced form of MC cell death may be ferroptosis. GO analysis was also shown in Figure 4D.

### Metabolomics analysis in MC cells treated by FZNZ

**FIGURE 5:**
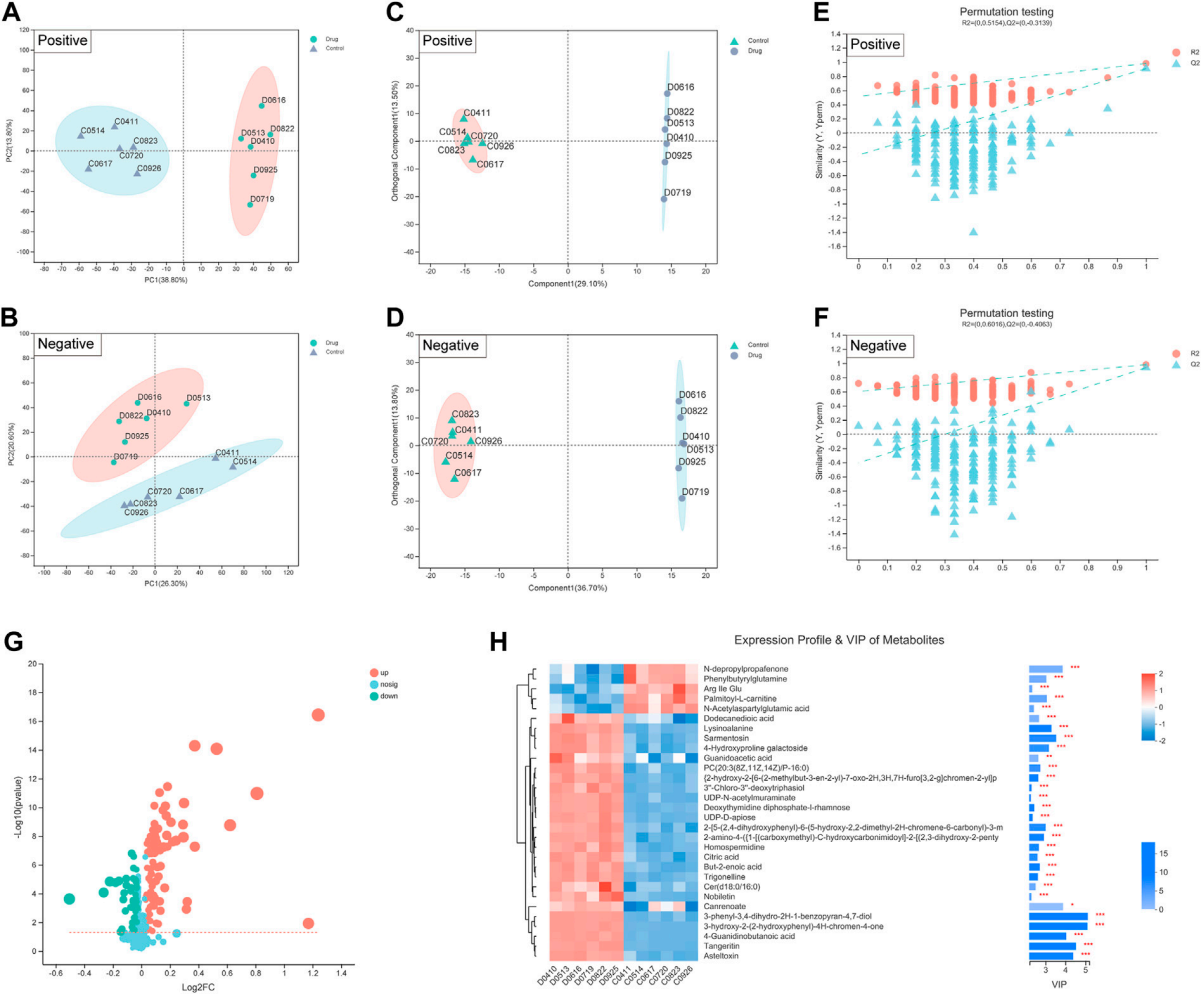
Biomarkers identification after FZNZ treated in MC cells by metabolomics. Principal component analysis, each point represents a sample, and ellipses represent 95% confidence regions (n = 6) in **(A)** positive ion mode; **(B)** negative ion mode; OPLS-DA score plots in **(C)** positive ion mode; **(D)** negative ion mode; Permutation testing in **(E)** positive ion mode; **(F)** negative ion mode; The slope of R2 is greater than 0, and the intercept of Q2 on the *Y* axis is less than 0.05, indicating that the model is effective. **(G)** Volcano map. The abscissa is the fold change value of the difference, and the ordinate is the statistical test value of the difference in the expression of metabolites. Each dot in the figure represents a metabolite, and the size of the dot represents the VIP value. **(H)** Heatmap of potential biomarkers. Red depicts that the expression of biomarker was increased, blue depicts that the expression of biomarker was decreased.

Additionally, metabolites are the end products of gene expression, and many intracellular life activities occur at the level of metabolites. To better reveal the differences between different groups of samples, we also adopted the PCA method. PCA showed a significant difference in the distribution of cellular metabolites between two groups (Figures 5A,B). The R2X (cum), R2Y (cum) and Q2 (cum) of the OPLS-DA model were obtained by seven-fold cross validation to verify the reliability of the model. Between two groups, the three parameters were 0.426, 0.998 and 0.934 in positive ion mode, while 0.505, 0.998 and 0.958 in negative ion mode (Figures 5C,D). Furthermore, the permutation testing method was used to verify the accuracy of the OPLS-DA model (Figures 5E,F). This indicated that the effect of FZNZ on MC cells was the main reason for the altered metabolomics.

We identified 134 differential metabolites with a threshold of VIP >1.0 and *p* < 0.05, which are shown in the differential volcano map (Figure 5G). Combined with the cluster heatmap and VIP bar chart, the expression pattern of metabolites in each sample, the *p*-value of metabolites in VIP and unidimensional statistics of multivariate statistical analysis were displayed to visually see the importance of different metabolites and the trend change of their expression (Figure 5H). Next, we explored the effect of specific metabolic pathways on the response to FZNZ therapy. HMDB compound classification and pathway enrichment were performed to determine the function of metabolites (Supplemental Figure S1).

### FZNZ induced ferroptosis integrated metabolite profiling and transcriptome analysis

**FIGURE 6:**
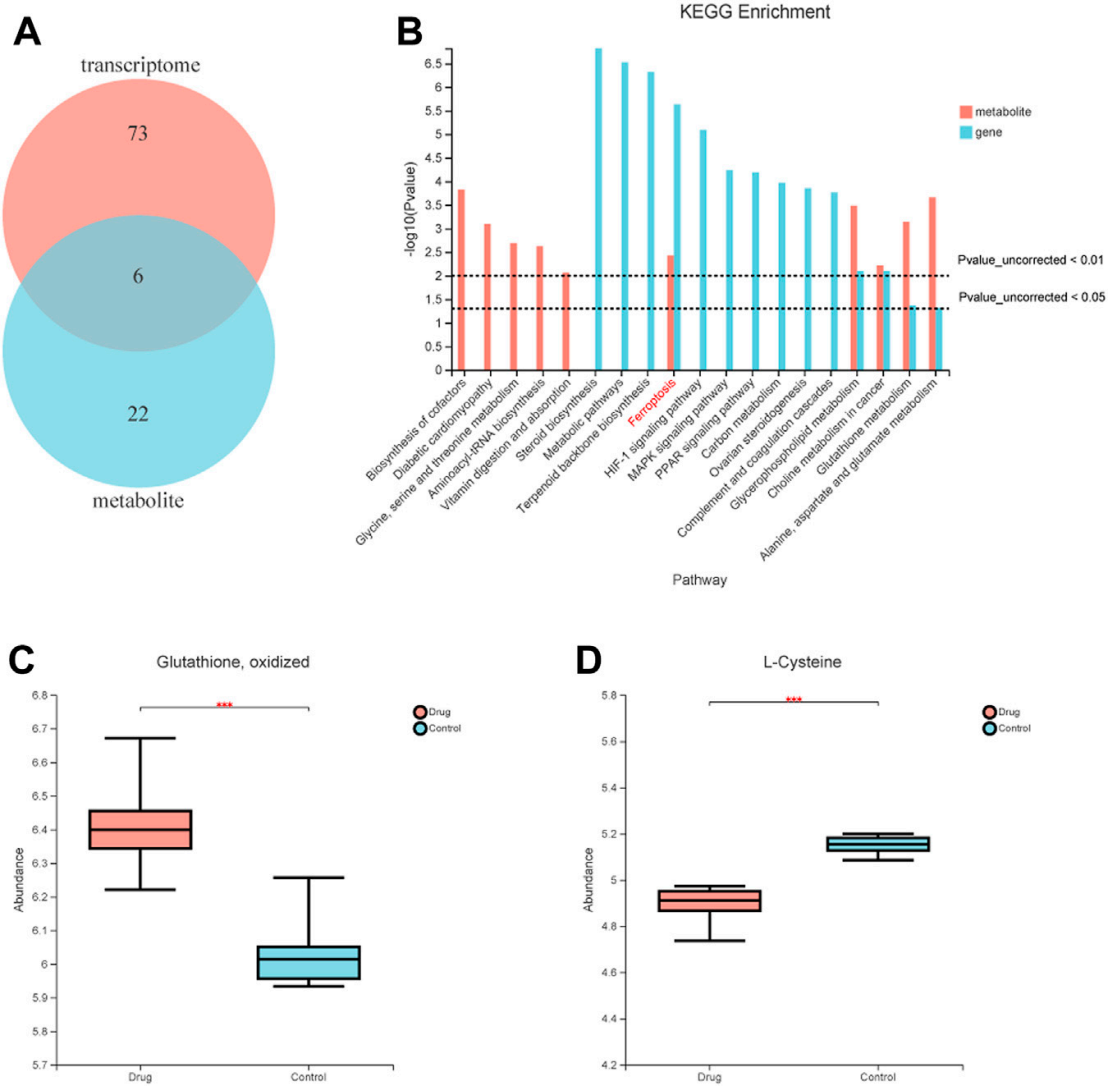
Correlations between transcriptomic and metabolome data of MC cells. **(A)** Venn diagram refers to the pathway enrichment analysis between transcriptomic and metabolome. **(B)** Integrative analysis of KEGG, which showed the most perturbed metabolic pathway in MC, ferroptosis. **(C)** The expression of oxidized glutathione in metabolome **(D)** The expression of Cysteine in metabolome.

To further understand the mechanism of FZNZ in GPL, we combined metabolomics and transcriptomics to compare and study the related biological issues at the metabolite and mRNA levels to uncover the key molecules and reveal the related mechanisms. KEGG pathway enrichment analysis refers to the enrichment analysis of selected differential gene sets and differential metabolic sets. We used the hypergeometric distribution algorithm to obtain pathways with significant enrichment of genes in the gene set and metabolites in the metabolite set. Six common pathways were considered significantly enriched when the *p*-value was <0.05 (Figure 6A). They were respectively Ferroptosis, Glycerophospholipid metabolism, Choline metabolism in cancer, Glutathione metabolism, Alanine, aspartate and glutamate metabolism and Mineral absorption (Figure 6B). We speculated that the FZNZ induced form of MC cells death may be related to ferroptosis and GSH metabolism. The associatd metabolite profiles were also specified in the metabolome assay. Oxidized glutathione (GSSG) showed an increasing trend, while cysteine showed a decreasing trend (Figures 6C,D). Studies have shown that cysteine is required for cell growth and protection against cell death (Liu et al., 2017). Cysteine depletion is an initial feature that triggers ferroptosis, which subsequently leads to depletion of intracellular glutathione (Zhang et al., 2021).

### Identification of potential biomarkers and pathways involved in ferroptosis

**FIGURE 7:**
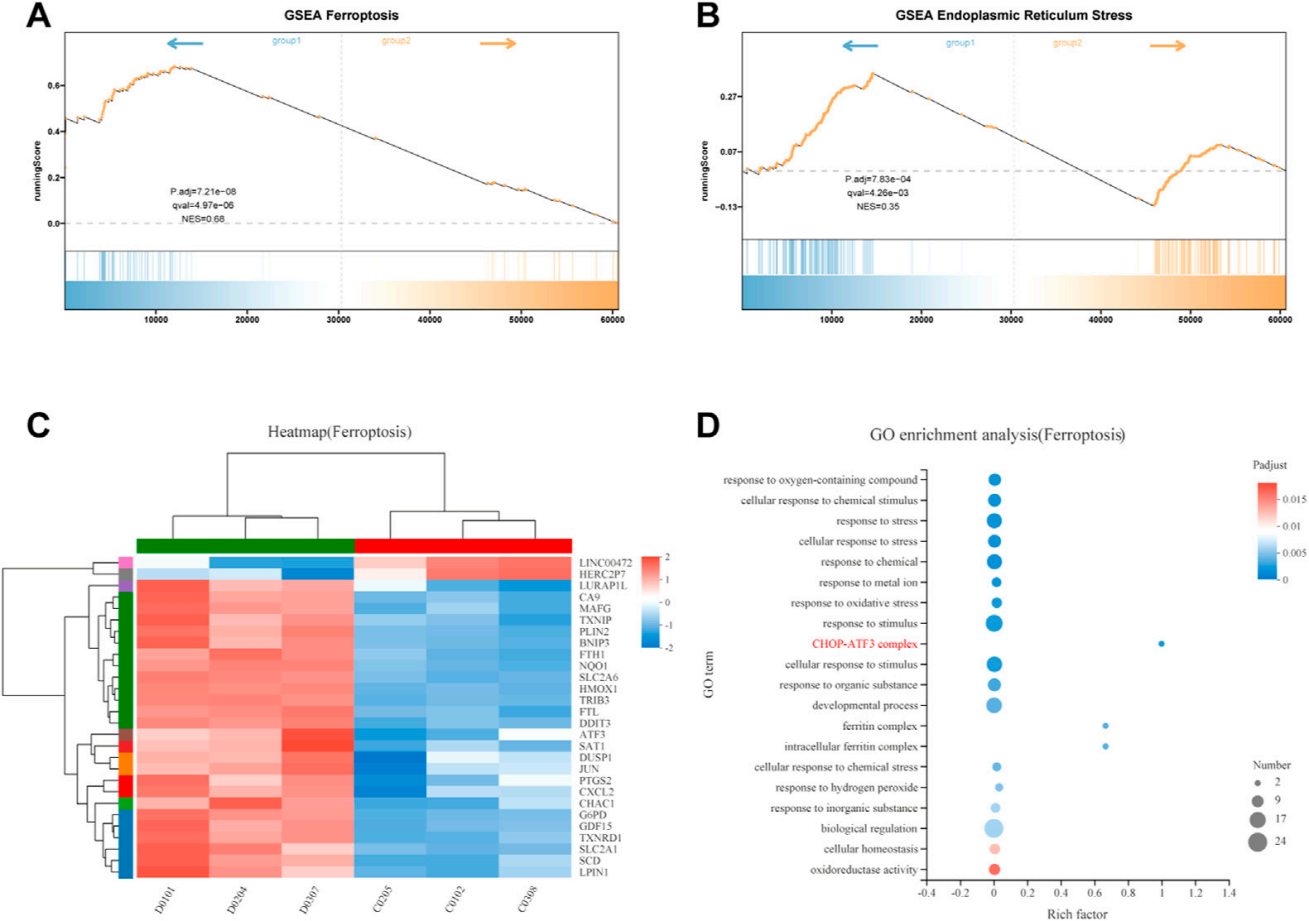
Integrative analysis of molecular signatures using transcriptomic and metabolomic data. **(A,B)** Results of GSEA, respectively, ferroptosis and response to endoplasmic reticulum stress **(C)** Heat map expression of ferroptosis related genes **(D)** GO analysis of ferroptosis related genes.

To elucidate the mechanism of FZNZ action on ferroptosis in MC cells, we performed Gene Set Enrichment Analysis (GSEA) to screen for differential pathways and related gene expression. FZNZ could regulate ferroptosis and the response to endoplasmic reticulum stress-associated genes identified by GSEA (Figures 7A,B). To further narrow the range of important target genes, we constructed a set of drug-associated ferroptosis genes. The regulatory effect of the key genes in the process of ferroptosis was checked in the FerrDb database (http://www.zhounan.org/ferrdb) (Zhou and FerrDb, 2020). The 28 ferroptosis genes were analyzed by heatmap (Figure 7C) and GO enrichment (Figure 7D). The CHOP-ATF3 complex had the highest Rich factor in GO enrichment analysis. RNA sequencing analysis also showed that FZNZ increased the mRNA levels of CHOP and ATF3 in MC cell lines. The upregulation of the ER stress response gene ATF3 serves as a useful pharmacodynamic marker of ferroptosis. In addition, it has been reported that CHAC1 is regulated by ATF3. CHAC1 degrades GSH and plays a role between ER stress and ferroptosis, which is not only induced in response to ER stress but also a positive regulatory marker of ferroptosis. We hypothesized that FZNZ may induce ER stress while inducing ferroptosis, and this effect was related to ATF3/CHOP/CHAC1.

### FZNZ promotes ferroptosis in MC cells

**FIGURE 8:**
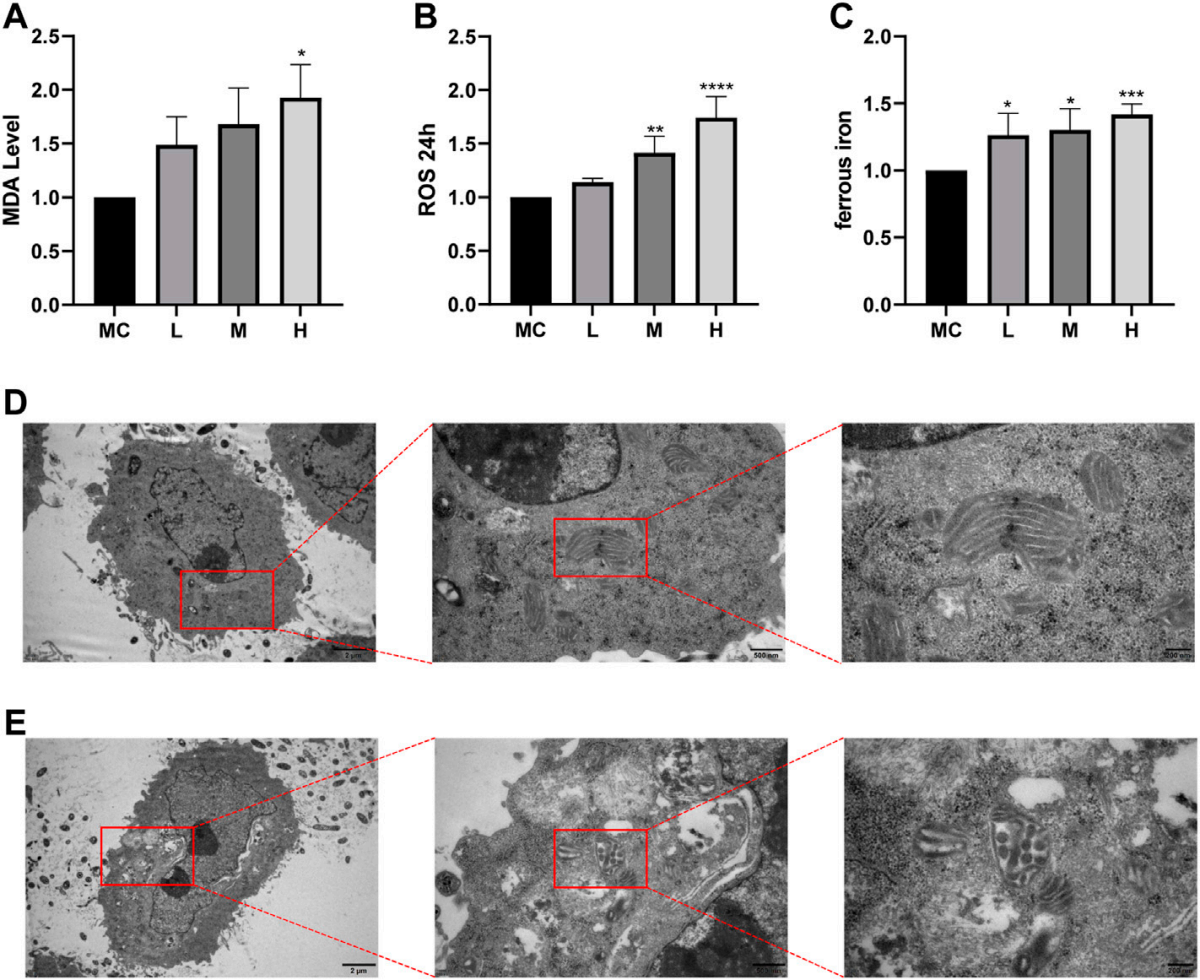
FZNZ induced ferroptosis in MC cells. **(A)** Detection of lipid peroxidation by MDA assay kit; protein concentration was used for calibration. **(B)** Detection of the ROS in MC cells by black 96 plates from each group; cell counts were used for calibration. **(C)** Levels of intracellular iron were detected by black 96 plates after incubating with Fe2+ ions fluorescent probe FerroOrange in MC cells. **(D,E)** The morphological changes of mitochondria were detected by transmission electron microscopy and performed local magnification display. Data are shown as the mean ± SD; N = 3–5/group (*p < 0.05, **p < 0.01, ***p < 0.001).

Ferroptosis is driven by iron-dependent lipid peroxidation, so it is necessary to detect lipid peroxidation and the level of iron (Hu et al., 2021). Iron may also directly produce excessive ROS through the Fenton reaction, thus aggravating oxidative damage. In addition, mitochondria usually exhibit a shrunken, dense morphology during ferroptosis. To investigate whether the effect of FZNZ on MC cells is mediated by ferroptosis, we examined the levels of MDA, ROS, and ferrous iron for further validation. Interestingly, ROS and MDA are quite specific lipid peroxidation products that are usually defined as markers of ferroptosis. They tended to increase in response to the drug and were significantly different at high doses (Figures 8A,B). To further observe another feature of ferroptosis, the concentration of ferrous iron in the drug treatment group was significantly increased, as shown in Figure 8C. Next, the cells showed signs of ferroptosis, such as rupture of cell membranes, smaller cell mitochondria, increased membrane density, and reduced cristae in morphological observations after the addition of drugs (Figures 8D,E). These results indicated that FZNZ induced ferroptosis in MC cells *in vitro*, which confirmed the sequencing results.

### FZNZ negatively regulates ferroptosis by regulating glutathione metabolism

**FIGURE 9:**
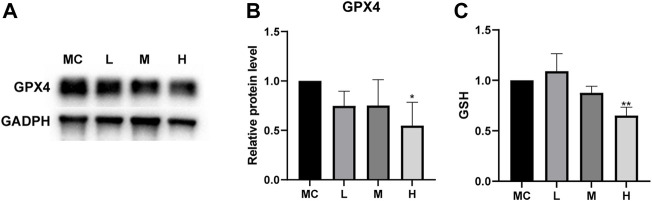
FZNZ regulated GPX4/SGH axis. **(A,B)** Western blot analysis of the expression of proteins GPX4; GAPDH was used as loading control. **(C)** Detection of the GSH content in MC cells with the treatment of FZNZ. Data are shown as the mean ± SD; N = 3–5/group (*p < 0.05, **p < 0.01, ***p < 0.001).

The combination of sequencing and analysis suggested that ferroptosis was also related to glutathione metabolism. GSH depletion and the loss of GPX4 activity are the main causes of ferroptosis associated with glutathione metabolism (Stockwell et al., 2017). GPX4 is an antioxidant enzyme that converts potentially toxic lipid hydroperoxides to nontoxic lipid alcohols using GSH as a cofactor (Yang et al., 2014), and the inactivation of GPX4 can ultimately cause cell death (Linkermann et al., 2014). We measured the expression of GPX4 in cells after drug treatment. Western blotting showed that the protein expression was decreased in MC cells after drug treatment compared to the normal control group (Figures 9A,B). Cellular GSH efflux can sensitize cells to ferroptotic agents (Cao et al., 2019). Under the action of a high dose of FZNZ, the level of reduced GSH showed a downward trend (Figure 9C). These results suggested that FZNZ influenced the GPX4/GSH reduction system in MC cells.

### FZNZ induced the ER stress-related ferroptosis in MC cells

**FIGURE 10:**
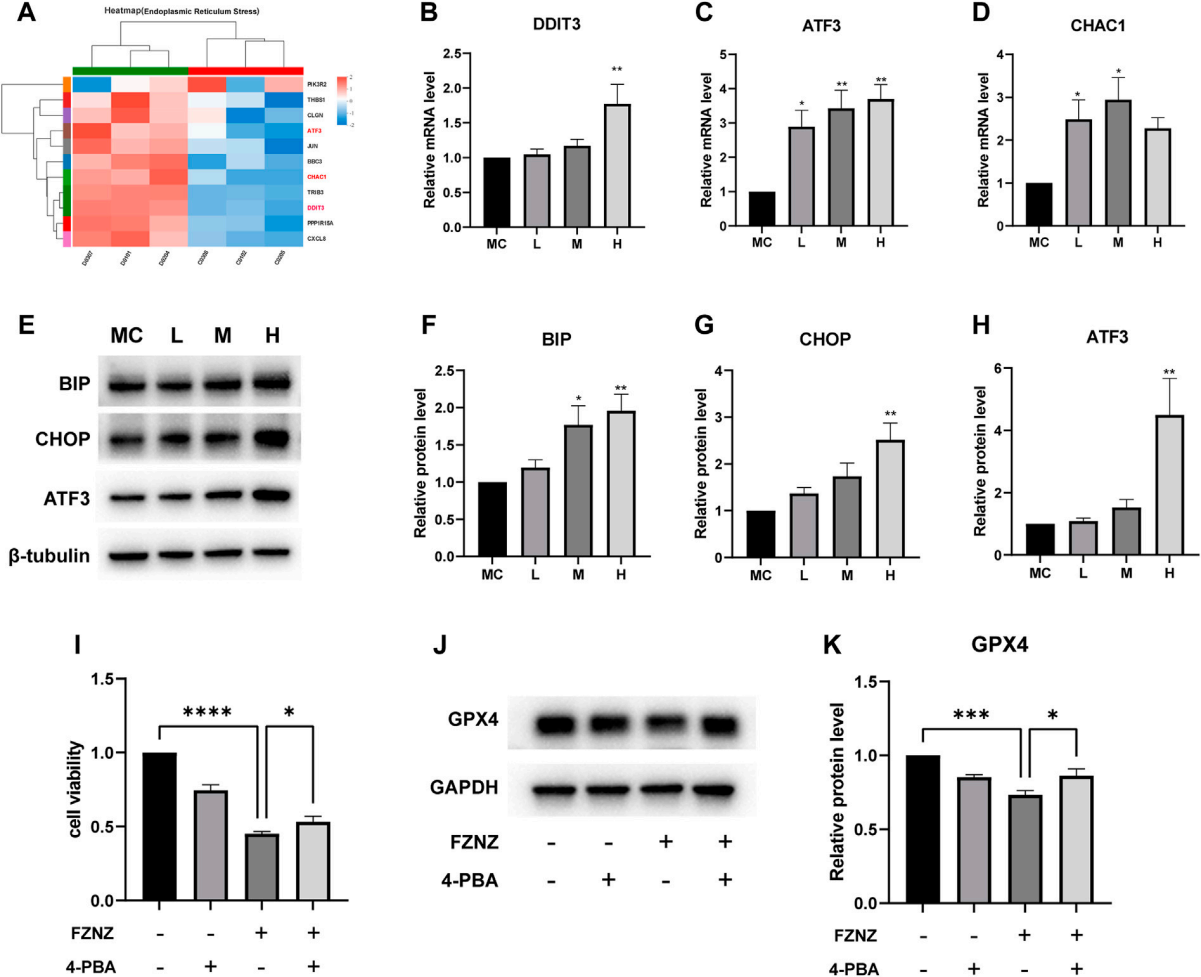
FZNZ induced the ER stress-related ferroptosis in MC cells **(A)** Heat map of 11 ER stress-related genes in RNA-seq. **(B–D)** mRNA level of DDIT3, ATF3 and CHAC1, respectively. **(E–H)** Expressions of ER stress-related proteins were measured by western blot; β-tubulin was used as loading control. ER stress inhibitor 4-PBA (2.5 mM) and/or high dose FZNZ were added in MC cells for 24 h. Cell viability was determined by CCK8 assay **(I)**. The protein of ferroptosis marker GPX4 was measured by western blot; GAPDH was used as loading control **(J,K)**. Data are shown as the mean ± SD; N = 3–5/group (*p < 0.05, **p < 0.01, ***p < 0.001).

To verify the correlation between ferroptosis and ER stress predicted by bioinformatics (Figure 7B). We constructed an ER stress gene set from datasets M10844 and MA25383 in MSigDB (https://www.gsea-msigdb.org/gsea/msigdb/). A heatmap was used to show the upregulation and downregulation of genes in sequencing according to 11 ER stress related gene (Figure 10A). Among these genes, we also found the ferroptosis related ATF3/CHOP complex predicted in GO enrichment (Figure 7D). It has been reported that the transcription factor ATF3 could regulate mRNA induction of CHAC1 (Crawford et al., 2015). Although the precise molecular mechanisms of ferroptosis are being intensively studied, the upregulation of CHAC1 is widely accepted as an early ferroptosis marker that also contributes to the progression of GSH degradation (Mungrue et al., 2009) and ferroptosis execution (Dixon et al., 2014). Then we evaluated whether this was also the case with MC cells. The results showed that the mRNA levels of ATF3/CHOP/CHAC1 all presented an increasing trend both in sequencing and qRT‒PCR (Figures 10B–D). Subsequently, we examined whether there was ER stress and detected related protein. BIP, an ER molecular chaperone, can recognize misfolded proteins in the ER and is therefore considered a marker of ER stress (Pobre et al., 2019). We detected its protein level to determine the occurrence of ER stress. At the same time, the protein level of ATF3/CHOP also showed an upward trend, which also verified the occurrence of ER stress (Figures 10E–H).

We also combined ER stress inhibitor 4-Phenylbutyric acid (4-PBA) (Ma et al., 2020) treatment with FZNZ in MC cells to estimate the role of ER stress in decoction induce ferroptosis. The effectiveness of combine treatment through measured cell viability using CCK-8 kit. As shown in Figure 10I, the cell viability was increased in combination with 4-PBA, which could alleviate the damage of FZNZ. In parallel, we examined the downstream ferroptosis marker GPX4 and found that 4-PBA could increase FZNZ-induced downregulation of GPX4 (Figures 10J,K).

## Discussion

The prevalence of GPL is a serious medical problem that plagues people worldwide (Liu et al., 2019). Without prompt treatment, GPL can turn into gastric cancer (Han et al., 2019). Preliminary clinical trials by our group have shown that FZNZ can significantly improve the clinical symptoms of GPL, especially in patients with dysplasia (Xin et al., 2021a). FZNZ could significantly improve the pathological score of gastric mucosae and improve the clinical manifestations of patients with stomach distension and pain (Xin et al., 2021b). Due to the complexity of FZNZ and the limitation of theories, the specific pharmacological mechanism was not completely clear, so we further studied the mechanism of FZNZ.

Metabolomics, as an important component of systems biology, can reveal the metabolic characteristics of the whole system after intervention, which is in line with the general concept of TCM (Wang et al., 2015; Shi et al., 2016). Meanwhile, transcriptomics studies the expression of gene, which might be an important means to study cell phenotype and function. Our study combined the two omics to explore the underlying mechanism and potential biomarkers of TCM, which might help to confirm the safety and efficacy of TCM and is conducive to modern research. We used MNNG-induced GES-1 cells as a precancerous model and performed multi-omics detection with corresponding bioinformatics method. It was found that FZNZ might induce the death of MC cells by inducing ferroptosis. We then verified that FZNZ promoted lipid peroxidation and mitochondrial damage, increased ferrous iron and ROS levels, and diminished GSH levels. Mechanistically, GSEA suggested that the occurrence of ferroptosis may be related to glutathione metabolism and ER stress.

The ER is a central hub that drives the lipid peroxidation that promotes cell death through ferroptosis. We found that FZNZ could induce ER stress (the overexpression of BIP) and ER stress participated in the occurrence of ferroptosis in the recovery experiment. In addition, we retrieved ferroptosis-related genes from the FerrDb database and cross-tabulated them with transcriptomic differential gene analysis for GO analysis and identified ATF3-CHOP as a key ferroptosis-related complex. ATF3 has been shown to be involved in oxidative stress (Hai et al., 1999), GSH metabolism (Wang et al., 2020) and cancer (Gargiulo et al., 2013). The ATF3-CHOP axis can activate CHAC1 at the transcriptional level (Hamano et al., 2020). A previous study showed that the expression level of CHAC1 was associated with ER stress induction (Gagliardi et al., 2019). Increased CHAC1 expression levels are widely recognized as an indicator of early ferroptosis and are associated with GSH degradation and the initiation of ferroptosis. These results were consistent with the observations of the present study. In drug-treated MC cells, both the mRNA and protein levels of ATF3 were elevated in response to FZNZ, and both its downstream regulatory genes DDIT3 and CHAC1 were also elevated. We observed that CHAC1 overexpression was accompanied by an increase in intracellular lipid peroxide levels and a decrease in GPX4 protein levels, leading to the induction of ferroptosis. Reduced GPX4 activity leads to the accumulation of lipid peroxides and oxidative damage, resulting in ferroptotic cell death (Stockwell, 2022). Other mechanisms controlling the degradation of GPX4 also modulate sensitivity to ferroptosis (Wu et al., 2019). Thus, this study demonstrated for the first time that a TCM decoction induced ferroptosis in the treatment of GPL.

In this study, we suggested that FZNZ could be considered as a novel treatment strategy for GPL patients, although further investigation is needed because of some limitations in the present study. First, based on the results of this study, we can only show that FZNZ regulates ER stress and ferroptosis, but the detailed molecular mechanism of its internal regulation needs further investigation. The direct ligand of ER stress or ferroptosis related proteins could be explored by SPR or ITC in the future. Second, although the effectiveness of FZNZ has been confirmed *in vitro*, further animal experiments are needed. Fortunately, our previous clinical trial can also illustrate the effectiveness of FZNZ.

## Conclusion

In summary, these findings indicated that FZNZ could induce ferroptosis and ER stress in MC cells, which may be due to crosstalk with the ATF3/CHOP axis. This study is the first report of ferroptosis induction through traditional Chinese medicine compound in GPL cells. Our study combined bioinformatics with experiments to explore the molecular mechanism of FZNZ in the treatment of GPL. Based on these investigations, we propose that FZNZ deserves further exploitation as a promising compound for the discovery of novel anti-GPL drugs.

## Data Availability

The datasets presented in this study can be found in online repositories. The names of the repository/repositories and accession number(s) can be found in the article/Supplementary Material.
