# Supplementary figures and images for: Fuzheng Nizeng Decoction regulated ferroptosis and endoplasmic reticulum stress in the treatment of gastric precancerous lesions: A mechanistic study based on metabolomics coupled with transcriptomics
Source: Front Pharmacol. 2022 Nov 23;13:1066244. doi: 10.3389/fphar.2022.1066244 (PMC9727497; doi:10.3389/fphar.2022.1066244)

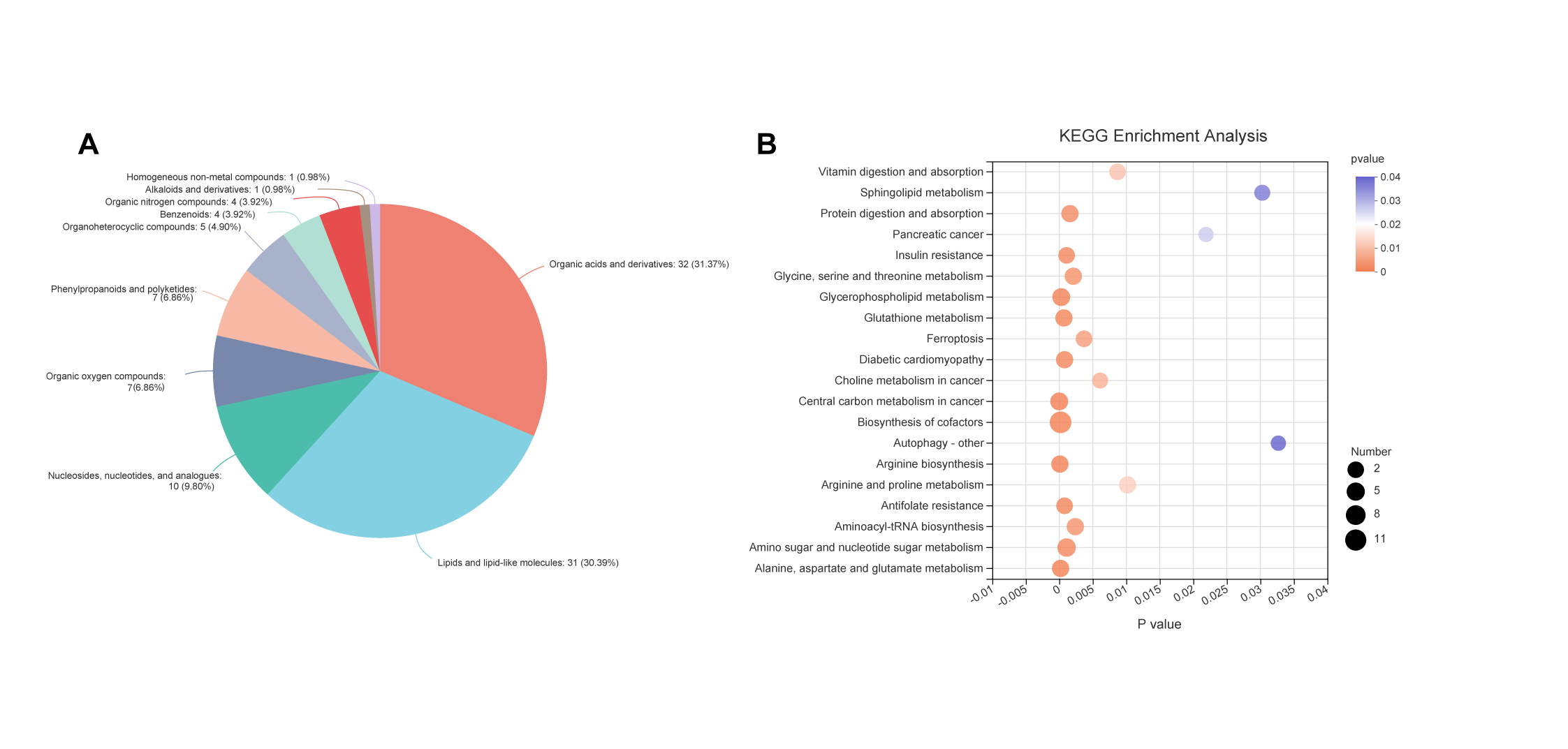

Supplement: Supplementary file 1 [file Image1.TIF]
