# Supplementary material for: Fuzheng Nizeng Decoction regulated ferroptosis and endoplasmic reticulum stress in the treatment of gastric precancerous lesions: A mechanistic study based on metabolomics coupled with transcriptomics
Source: Front Pharmacol. 2022 Nov 23;13:1066244. doi: 10.3389/fphar.2022.1066244 (PMC9727497; doi:10.3389/fphar.2022.1066244)
Supplement: Supplementary file 2 [file DataSheet1.PDF]

GPX4 Repeat 1

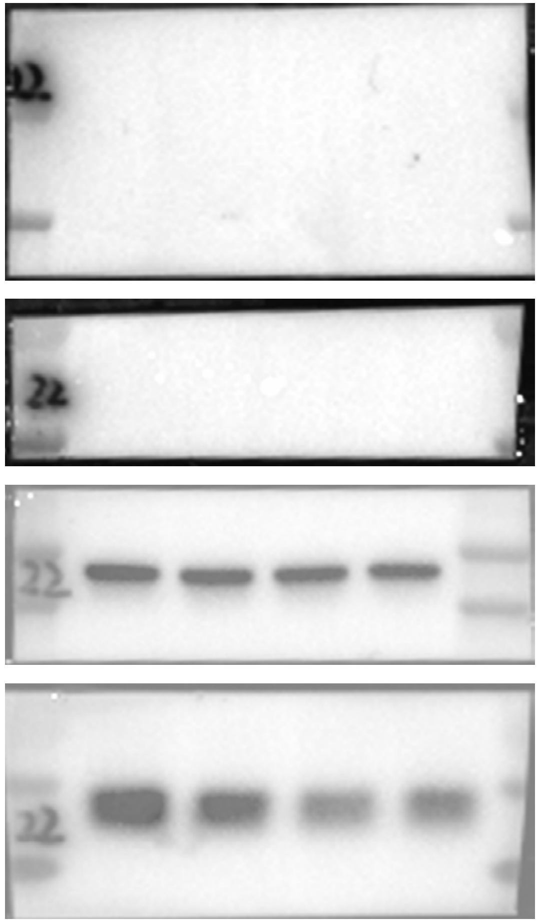

GPX4 Repeat 2

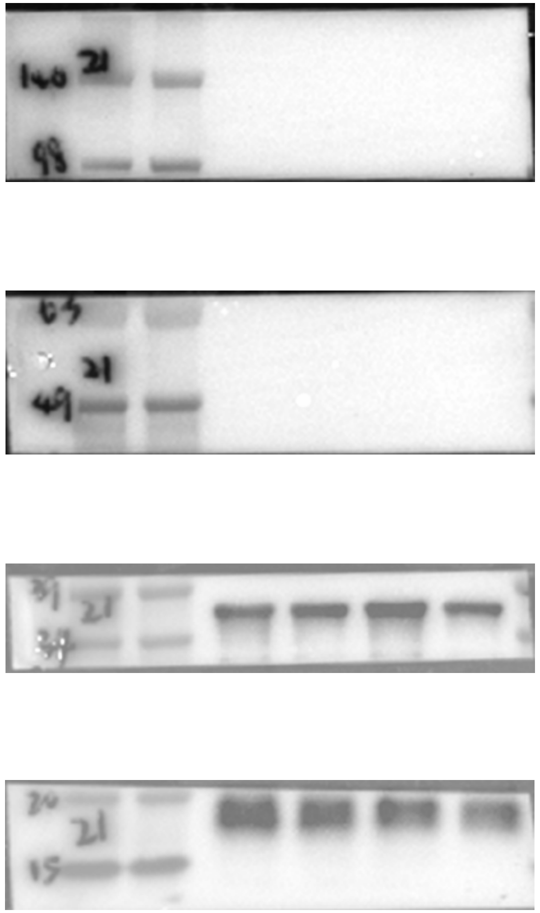

GAPDH(37KDa)

GPX4 (17KDa)

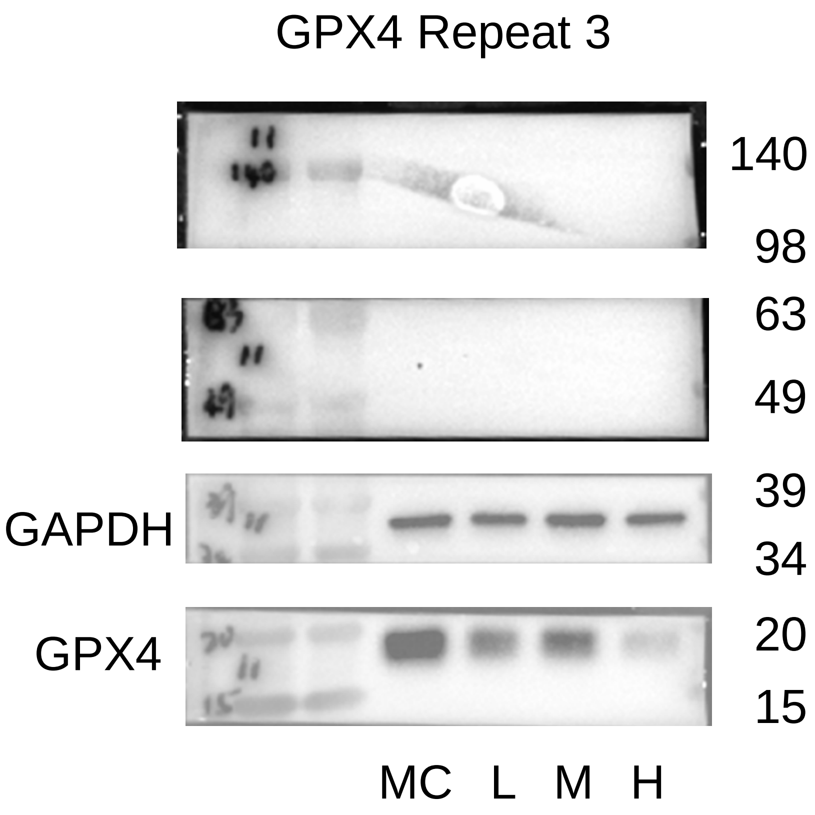

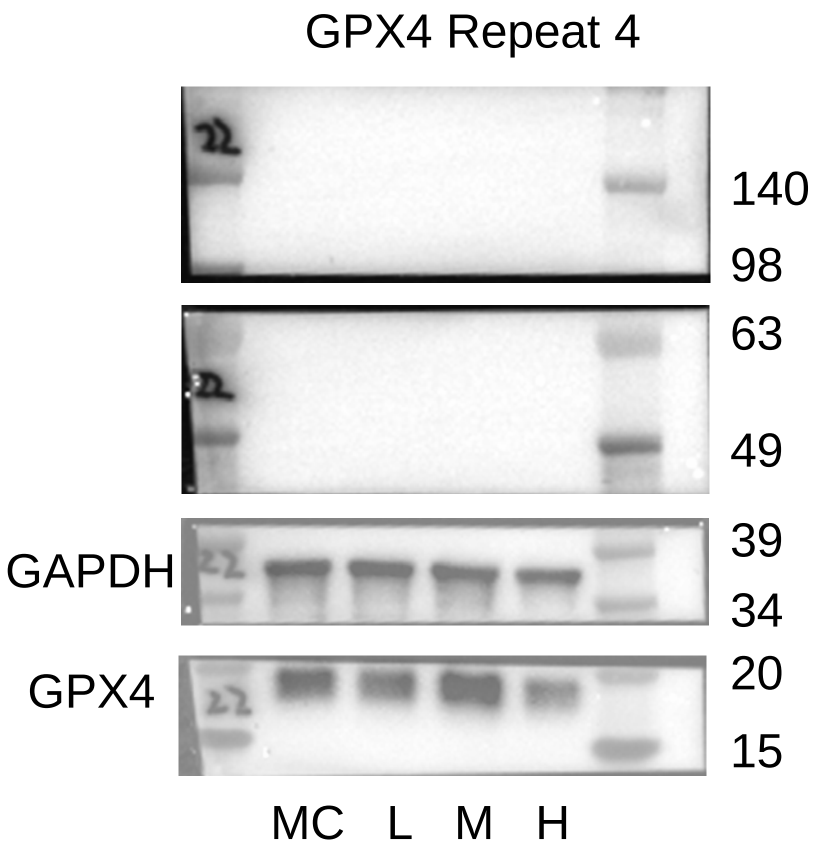

GAPDH(37KDa)

GPX4 (17KDa)

ATF3 Repeat 1

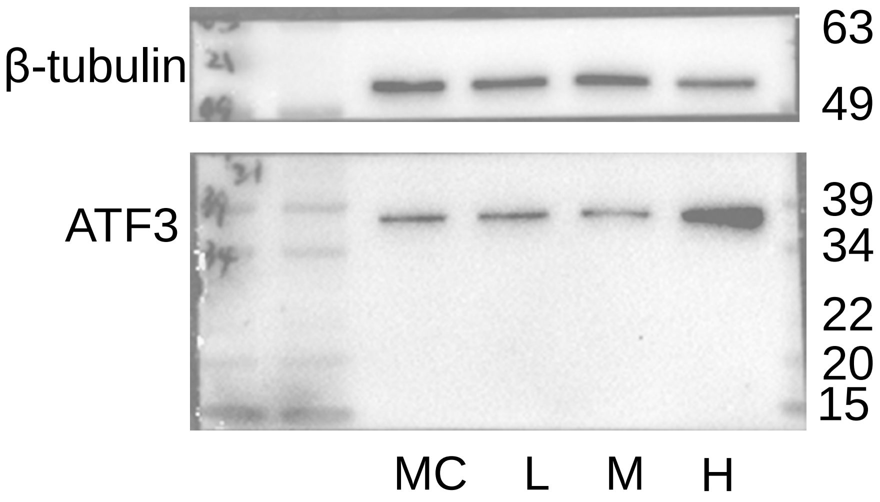

ATF3 Repeat 2

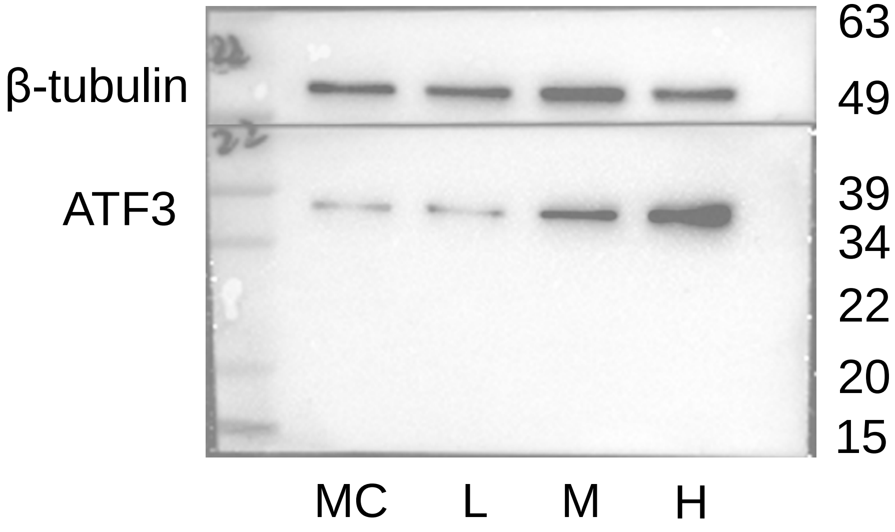

β-tubulin(55KDa)

ATF3 (34KDa)

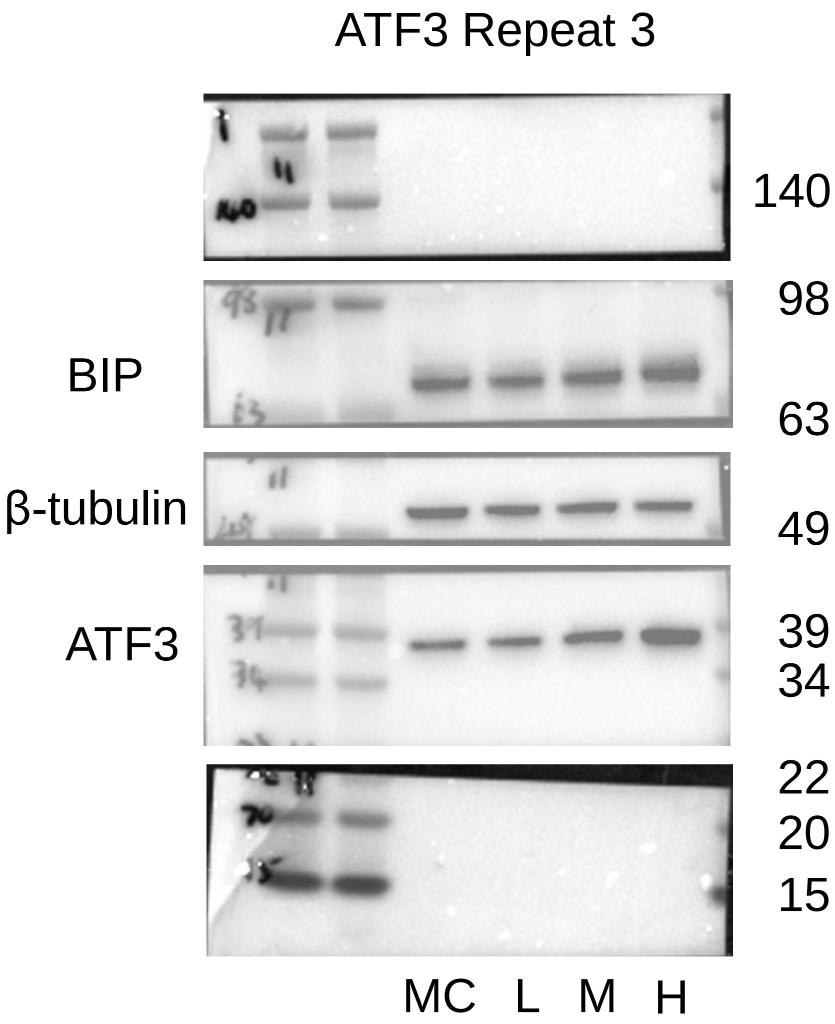

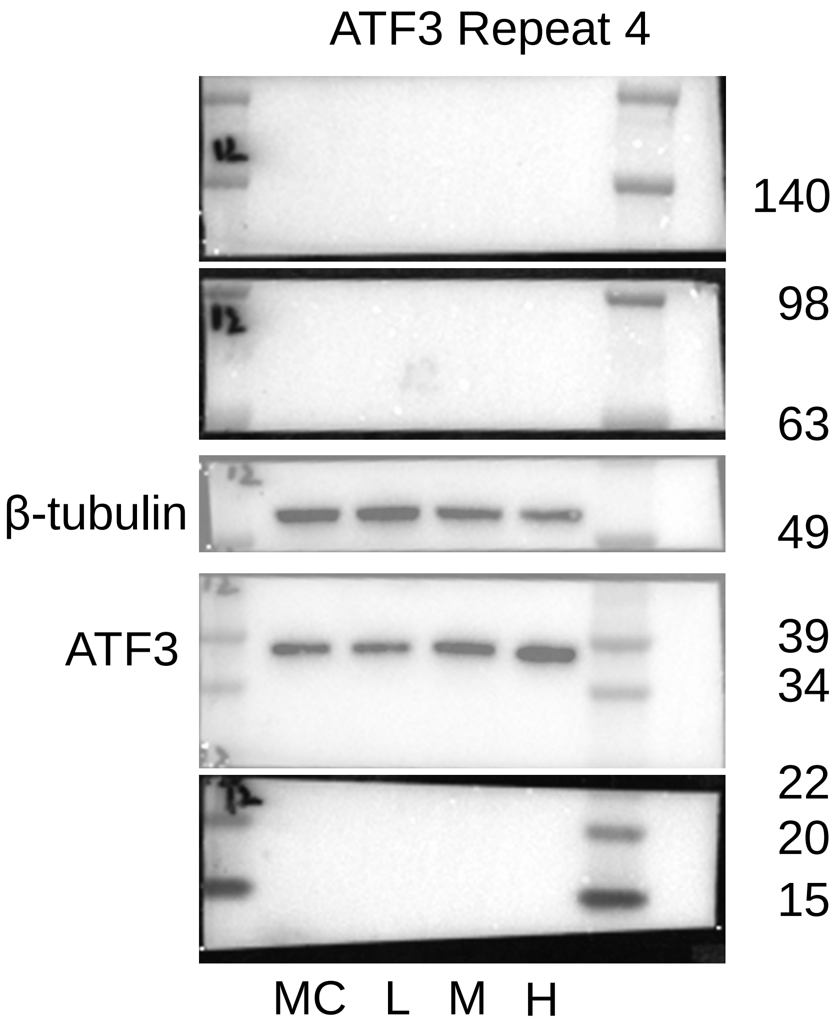

β-tubulin(55KDa)

ATF3 (34KDa)

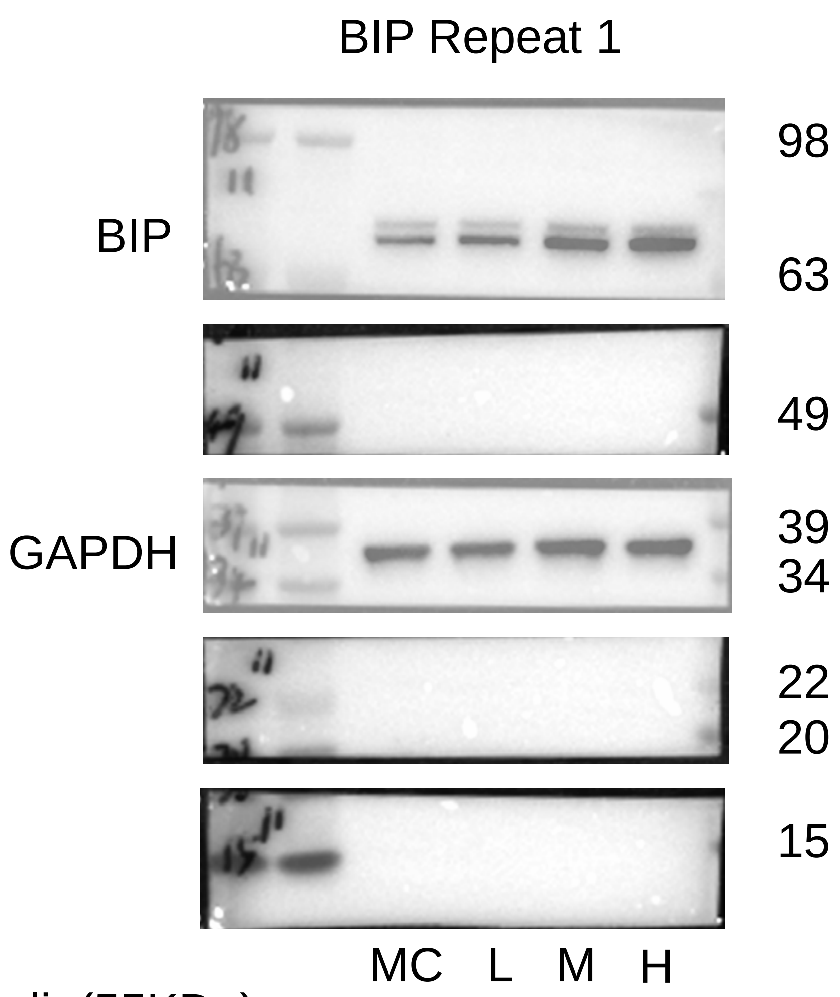

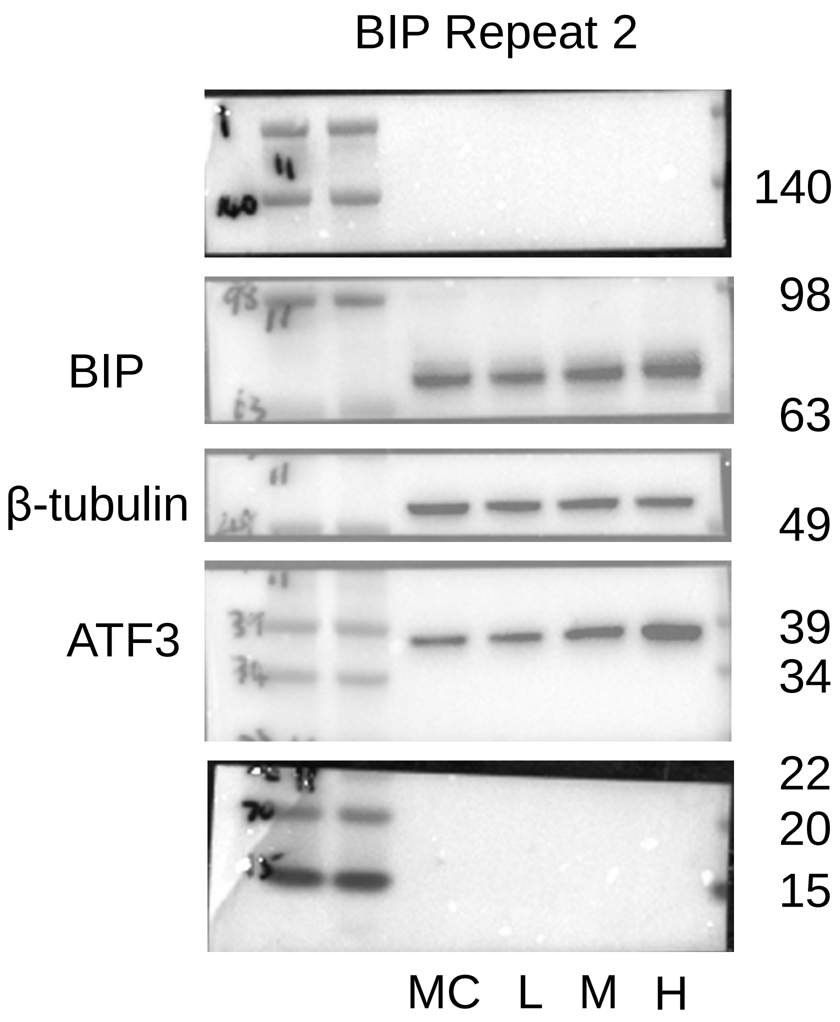

β-tubulin(55KDa)

GAPDH(37KDa)

BIP (80KDa)

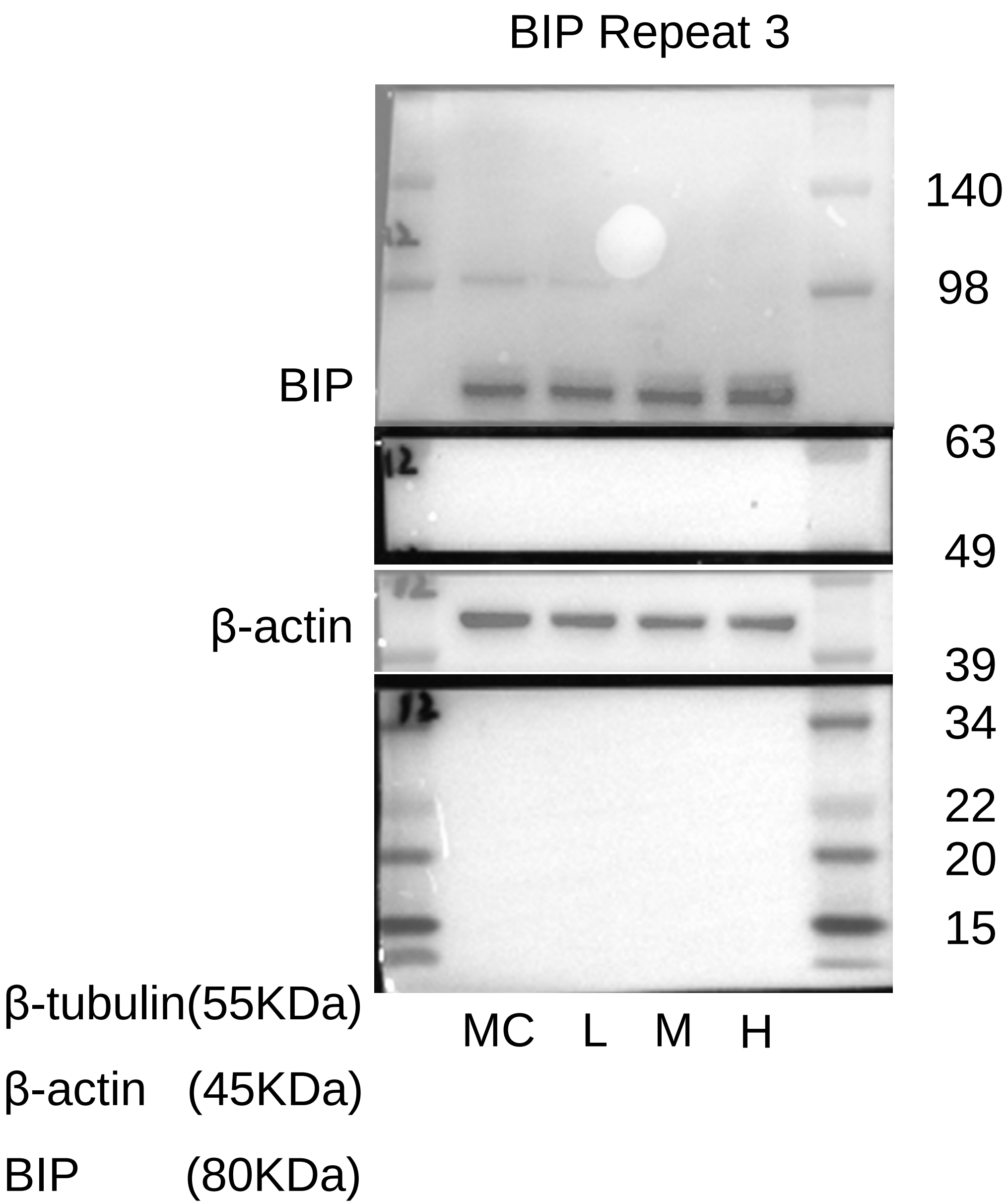
BIP Repeat 3
140
98
BIP
63
49
β-actin
39
34
22
20
15
β-tubulin(55KDa)
β-actin (45KDa)
BIP (80KDa)
MC L M H

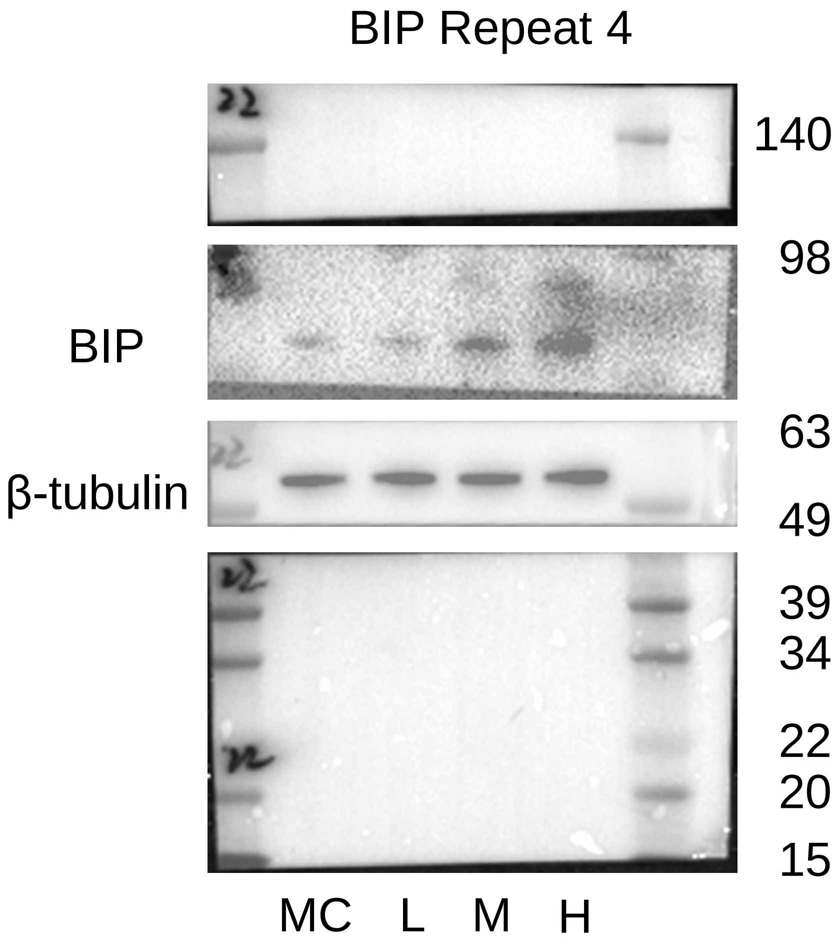
BIP Repeat 4
140
98
BIP
63
β-tubulin
49
39
34
22
20
15
MC L M H

CHOP Repeat 1

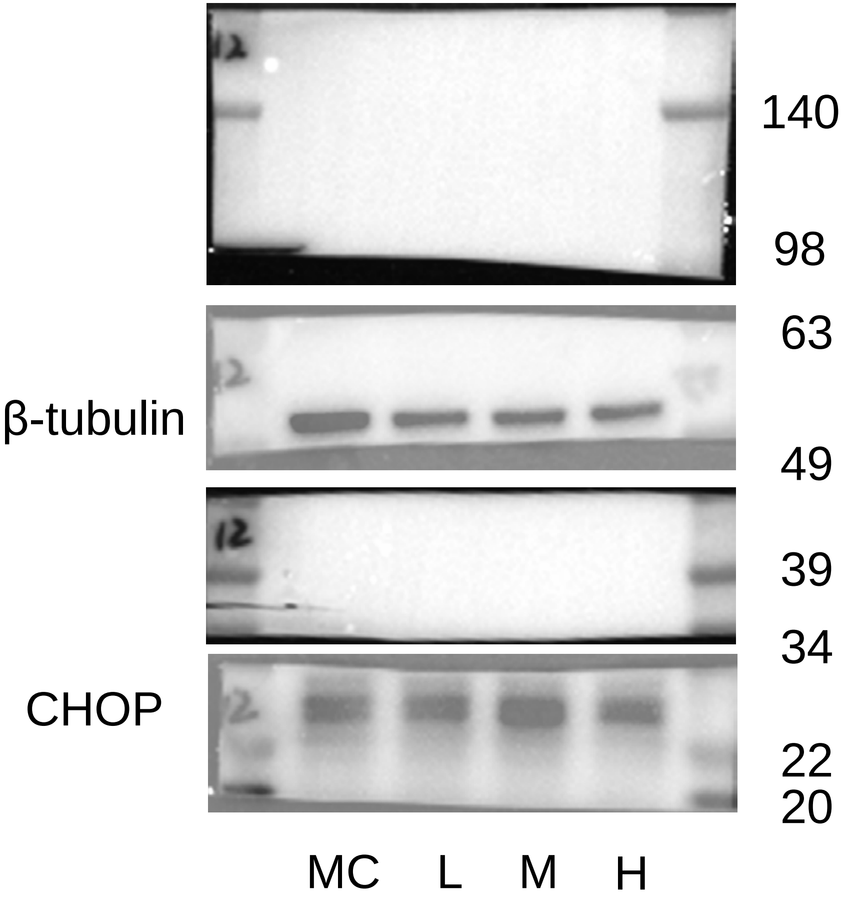

CHOP Repeat 2

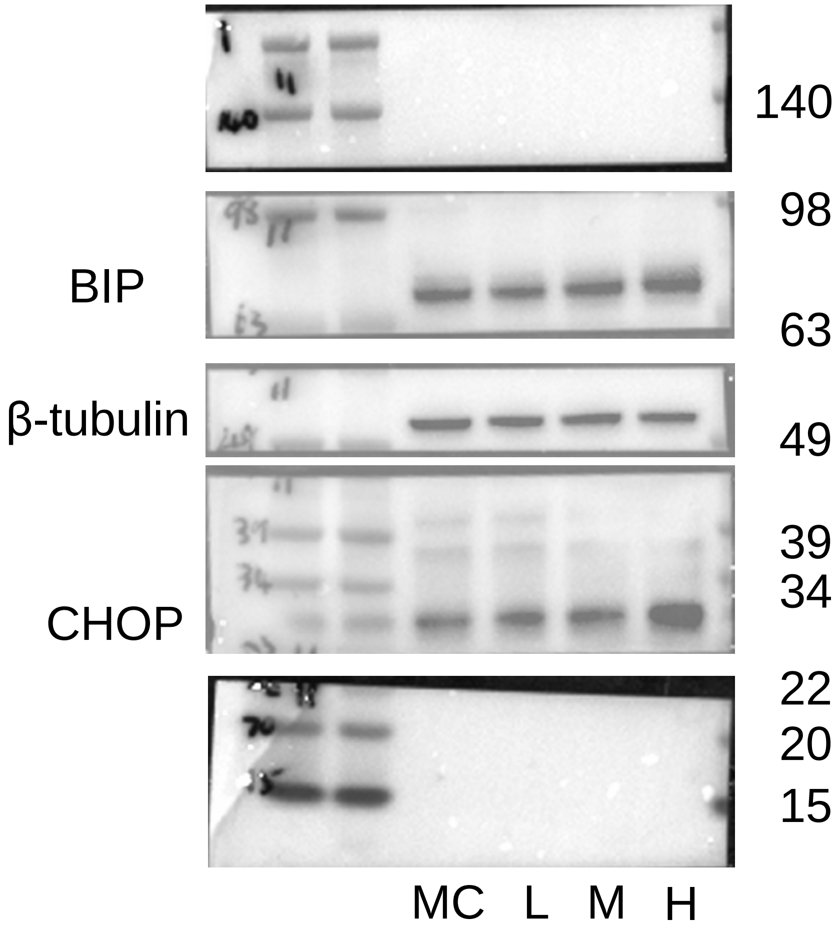

β-tubulin(55KDa)

CHOP (30KDa)

CHOP Repeat 3

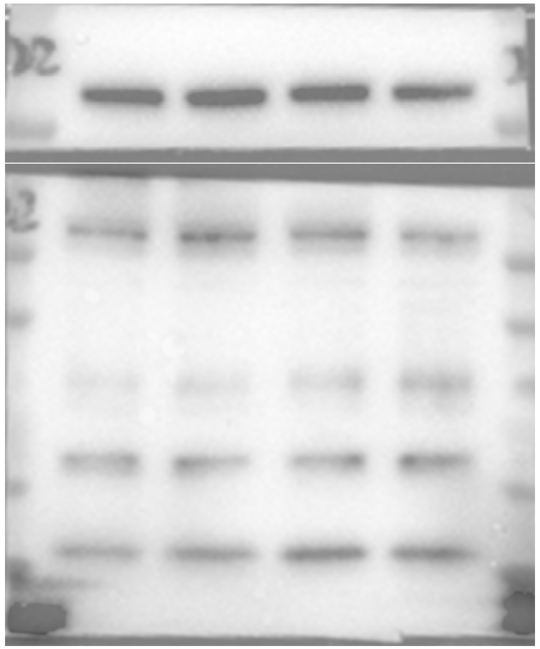

β-tubulin(55KDa)

CHOP (30KDa)

GPX4 Repeat 1

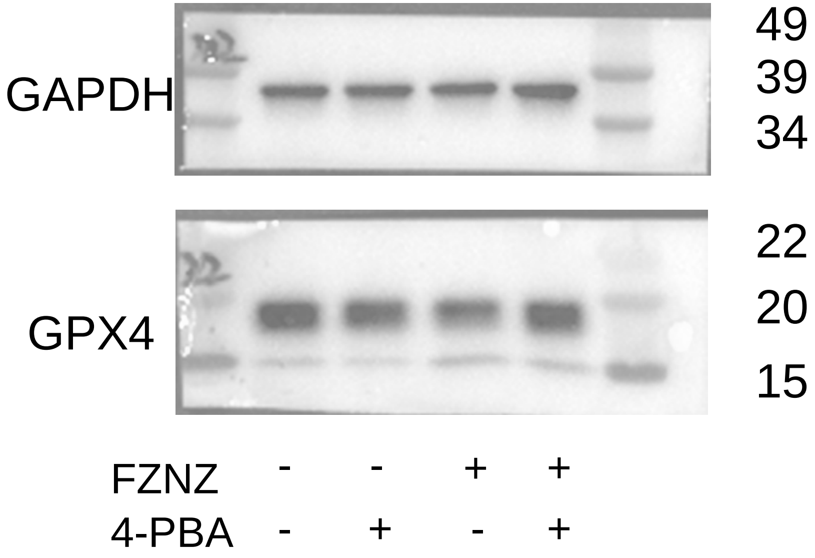

GPX4 Repeat 2

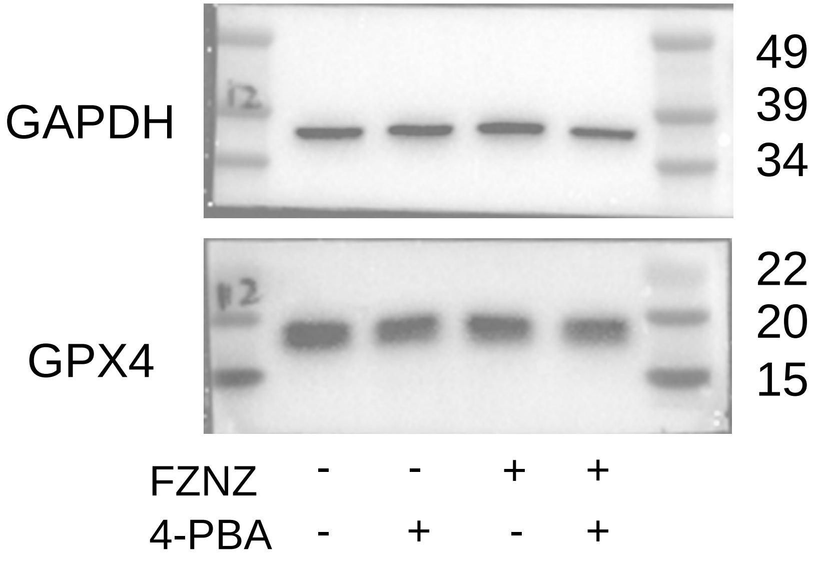

GPX4 Repeat 3

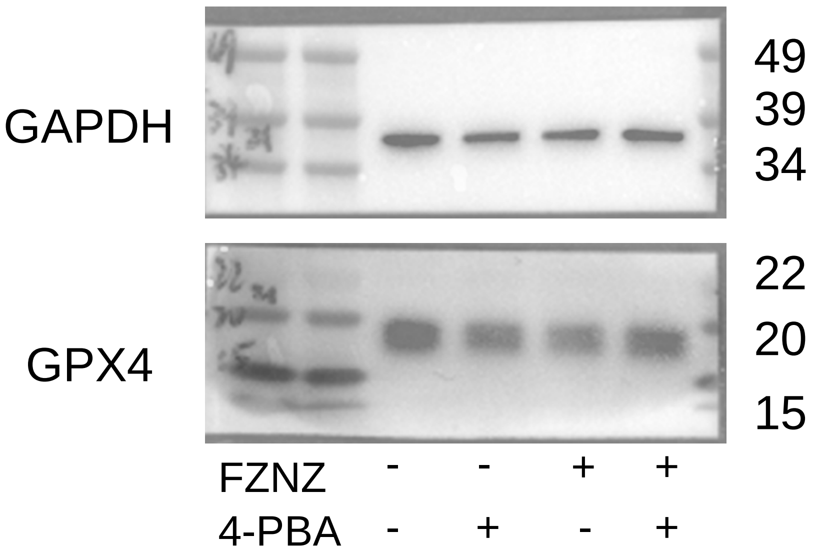

GAPDH(37KDa)

GPX4 (17KDa)
